# IGF2BP3 prevent HMGB1 mRNA decay in bladder cancer and development

**DOI:** 10.1186/s11658-024-00545-1

**Published:** 2024-03-19

**Authors:** Lei Lv, Qinqin Wei, Jianxiao Zhang, Yitong Dong, Zhenglei Shan, Na Chang, Ye Zhao, Po Bian, Qiyi Yi

**Affiliations:** 1 Department of Cancer Epigenetics Program, Anhui Cancer Hospital, The First Affiliated Hospital of USTC, Division of Life Sciences and Medicine, University of Science and Technology of China, Hefei, 230031 Anhui China; 2 Institute of Radiation Medicine, School of Basic Medical Sciences, Anhui Medical University, Hefei, 230032 Anhui China; 3 Medical Consulting Center, Hebei Children’s Hospital, Shijiazhuang, 050030 Hebei China; 4 The Second Clinical College, Anhui Medical University, Hefei, 230032 Anhui China; 5 Department of Radiation Oncology, The First Affiliated Hospital of USTC, Division of Life Sciences and Medicine, University of Science and Technology of China, Hefei, 230031 Anhui People’s Republic of China

**Keywords:** IGF2BP3, HMGB1, m6A, Methylation, Copy number amplification, Glycyrrhizin

## Abstract

**Background:**

IGF2BP3 functions as an RNA-binding protein (RBP) and plays a role in the posttranscriptional control of mRNA localization, stability, and translation. Its dysregulation is frequently associated with tumorigenesis across various cancer types. Nonetheless, our understanding of how the expression of the IGF2BP3 gene is regulated remains limited. The specific functions and underlying mechanisms of IGF2BP3, as well as the potential benefits of targeting it for therapeutic purposes in bladder cancer, are not yet well comprehended.

**Methods:**

The mRNA and protein expression were examined by RT-qPCR and western blotting, respectively. The methylation level of CpG sites was detected by Bisulfite sequencing PCR (BSP). The regulation of IGF2BP3 expression by miR-320a-3p was analyzed by luciferase reporter assay. The functional role of IGF2BP3 was determined through proliferation, colony formation, wound healing, invasion assays, and xenograft mouse model. The regulation of HMGB1 by IGF2BP3 was investigated by RNA immunoprecipitation (RIP) and mRNA stability assays.

**Results:**

We observed a significant elevation in IGF2BP3 levels within bladder cancer samples, correlating with more advanced stages and grades, as well as an unfavorable prognosis. Subsequent investigations revealed that the upregulation of IGF2BP3 expression is triggered by copy number gain/amplification and promoter hypomethylation in various tumor types, including bladder cancer. Furthermore, miR-320a-3p was identified as another negative regulator in bladder cancer. Functionally, the upregulation of IGF2BP3 expression exacerbated bladder cancer progression, including the proliferation, migration, and invasion of bladder cancer. Conversely, IGF2BP3 silencing produced the opposite effects. Moreover, IGF2BP3 expression positively correlated with inflammation and immune infiltration in bladder cancer. Mechanistically, IGF2BP3 enhanced mRNA stability and promoted the expression of HMGB1 by binding to its mRNA, which is a factor that promotes inflammation and orchestrates tumorigenesis in many cancers. Importantly, pharmacological inhibition of HMGB1 with glycyrrhizin, a specific HMGB1 inhibitor, effectively reversed the cancer-promoting effects of IGF2BP3 overexpression in bladder cancer. Furthermore, the relationship between HMGB1 mRNA and IGF2PB3 is also observed in mammalian embryonic development, with the expression of both genes gradually decreasing as embryonic development progresses.

**Conclusions:**

Our present study sheds light on the genetic and epigenetic mechanisms governing IGF2BP3 expression, underscoring the critical involvement of the IGF2BP3-HMGB1 axis in driving bladder cancer progression. Additionally, it advocates for the investigation of inhibiting IGF2BP3-HMGB1 as a viable therapeutic approach for treating bladder cancer.

**Graphical Abstract:**

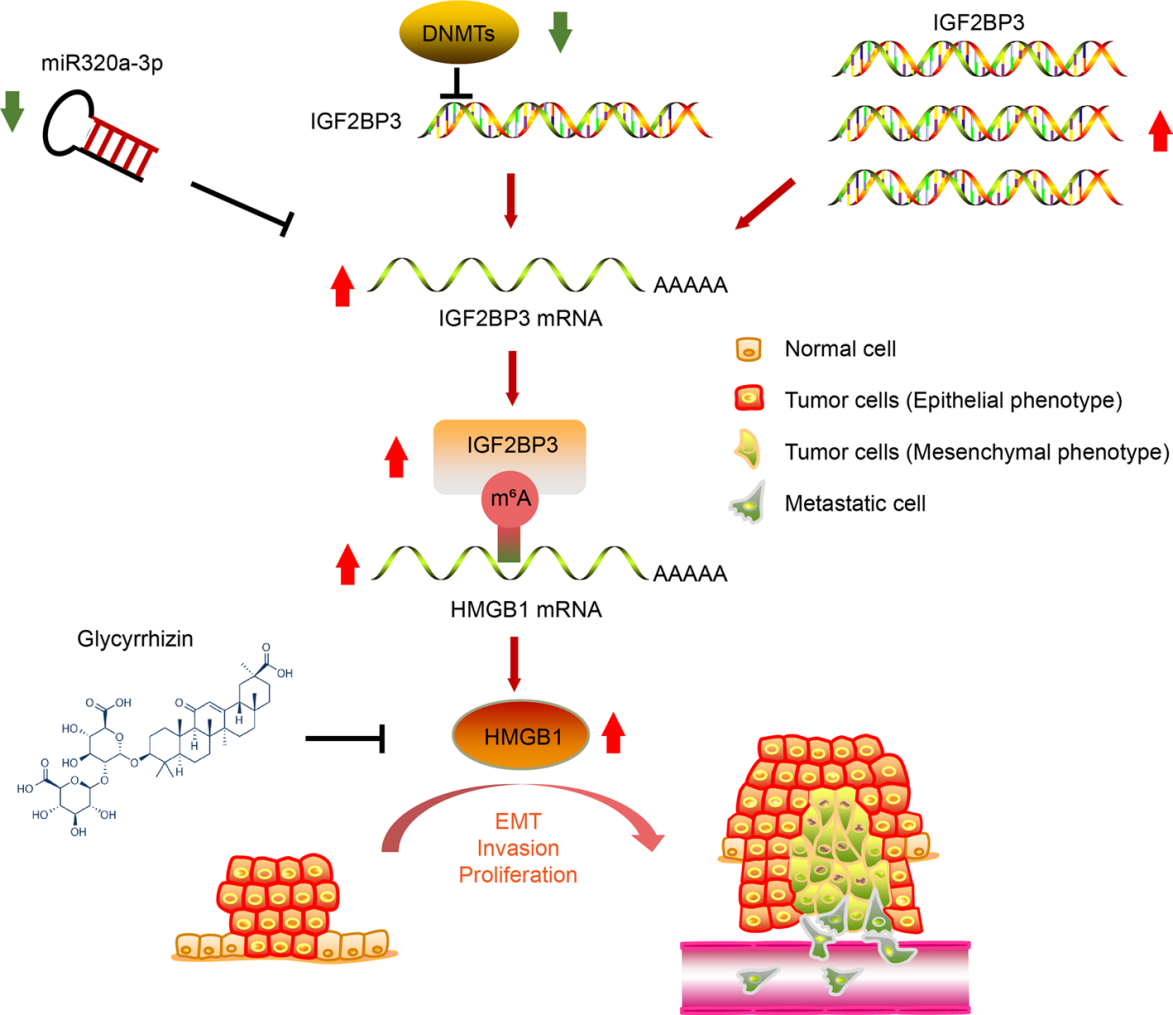

**Supplementary Information:**

The online version contains supplementary material available at 10.1186/s11658-024-00545-1.

## Background

In 2018, there were 549,393 new cases of bladder cancer reported, making it the second most common cancer in the genitourinary tract and the third highest cause of death among genitourinary tumors globally [1–3]. The development and progression of bladder cancer are influenced by various factors, including smoking, exposure to chemicals, and dysregulation of oncogenes and tumor suppressor genes [4]. Despite significant progress in the management of bladder cancer, the prognosis for high-risk patients remains unfavorable [5]. The progress in comprehending the biology of bladder cancer, along with extensive gene expression and sequencing initiatives, has resulted in recognition of the significance of genes with abnormal expression in the advancement of bladder cancer [6]. Based on this foundation, targeted therapy has emerged as a novel approach to treat individuals diagnosed with bladder cancer, particularly those in the advanced stages [7]. Hence, the identification of more efficient therapeutic targets is imperative in the treatment of bladder cancer.

N6-Methyladenosine (m6A) modification, a crucial mechanism in cancer development, is regulated by three classes of enzymes: writers (e.g., METTL3, METTL14, WTAP), erasers (e.g., FTO, ALKBH5), and readers (e.g., YTHDF1/2/3, IGF2BP1/2/3) [8, 9]. Writers facilitate the methylation process of mRNA and other nuclear RNA, whereas erasers function as demethylases for m6A. Readers decode the m6A modification details and engage in the translation and degradation of RNA molecules [10]. IGF2BP family, which includes IGF2BP1, IGF2BP2, and IGF2BP3, are m6A reader proteins. This family has been proven to increase the stability and translation of mRNA by binding to target mRNAs [11]. Among these, IGF2BP3 exhibits high expression in various tumor tissues, such as lung cancer [12], gastric cancer [13], pancreatic cancer [14], kidney cancer [15], hepatocellular carcinoma [16], breast cancer [17], and ovarian carcinoma [18], in contrast to adjacent normal tissues, indicating its role in promoting tumorigenesis [19]. Additionally, elevated levels of IGF2BP3 are indicative of the development of metastasis and a poor prognosis in renal cell carcinoma [20]. IGF2BP3 promotes aggressive phenotypes in colorectal cancer, lung tumorigenesis by affecting p53 stability, and recent reports suggest its involvement in bladder cancer cell proliferation [21–25]. Nevertheless, the precise functional significance of IGF2BP3 in the advancement of bladder cancer is still not well understood. And more importantly, the causes of aberrant IGF2BP3 expression in cancer is poorly understood.

This study investigates the significance of IGF2BP3 in bladder cancer from both clinical and biological perspectives. Our findings indicate that the expression of IGF2BP3 increases due to promoter hypomethylation, copy number gain/amplification, and a lack of miR320a-3p in bladder cancer. Moreover, elevated levels of IGF2BP3 are linked to enhanced proliferation, invasion, and unfavorable prognosis among individuals with bladder cancer. We further reveal that IGF2BP3 enhances the advancement of bladder cancer by stabilizing the mRNA of HMGB1. And glycyrrhizin, a known HMGB1 inhibitor, could potentially alleviate the malignant phenotype caused by the overexpression of IGF2BP3. These findings underscore the significance of the IGF2BP3-HMGB1 signaling pathway in the advancement of bladder cancer and propose it as a promising target for therapeutic intervention for bladder cancer.

## Methods

### Data acquisition in TCGA, oncomine, arrayexpress, and GEO

The clinical data, copy number alteration (CNA), DNA methylation, and RNA sequencing data of TCGA datasets were retrieved and downloaded from the UCSC xena (https://xenabrowser.net/datapages/) [26]. The “copy number (gene-level)—gistic2”, “DNA methylation—Methylation450k”, and “IlluminaHiSeq pancan normalized gene expression” were used in this study.

Furthermore, we conducted a thorough search for bladder cancer datasets that are accessible to the public on the oncomine (https://www.oncomine.org/), arrayexpress (https://www.ebi.ac.uk/arrayexpress/), and GEO (https://www.ncbi.nlm.nih.gov/geo/) platforms. We downloaded and utilized processed data from the one oncomine dataset "Sanchez-Carbayo bladder 2" [27], as well as two arrayexpress datasets (E-TABM-147 [28] and E-MTAB-4321 [29]), and seven GEO datasets (GSE48075 [30], GSE120736 [31], GSE1827 [32], GSE48276 [30], GSE32894 [33], GSE40355 [34], and GSE31684 [35]) for this study.

In addition, three GEO datasets related to embryonic development were also used in this study, including GSE65162 [36], GSE20954 [37], and GSE128419 [38].

### Survival analysis

Survival analysis, including OS (Overall survival), DSS (Disease specific survival), PFI (Progression free interval), and RFS (Recurrence free survival), were performed using Kaplan–Meier survival analysis of patients with bladder cancer. The analysis was conducted utilizing the R software packages called 'survival' and 'survminer'. By utilizing auto-selection of the optimal threshold, the individuals were divided into two categories based on their expression levels.

### Gene set enrichment analysis (GSEA)

GSEA was performed using the GSEAPreranked tool in GSEA software. The genes were ranked using the correlation coefficients between the expression of each gene and IGF2BP3. The “h.all.v7.2.symbols.gmt” was used as a reference gene set. A total of 1000 permutations were made. Pathways with FDR < 0.25 were considered enrichment significant [39].

### Analysis of immune cell infiltration

The correlation of IGF2BP3 expression level and abundance of immune cells, including B cells, macrophages, dendritic cells, neutrophils, CD4 + T cells, and CD8 + T cells, in bladder cancer was done using the TIMER (Tumor Immune Estimation Resource, https://cistrome.shinyapps.io/timer/).

### Human protein atlas (HPA)

The HPA (https://proteinatlas.org/) provides data on the mRNA and protein expression levels of human genes in both normal and tumor tissues [40]. Using HPA, we conducted a comparative analysis of IGF2BP3 mRNA and protein expression in both healthy bladder tissues and bladder cancer tissues.

### Cell culture, lentivirus transfection, and miRNA transfection

The bladder cancer cell lines RT112/84 (ECACC, Cat no: 85061106) and BFTC905 (BCRC, Cat no: 60068) cells were cultured in RPMI1640 medium (Biological Industries) and DMEM medium (Biological Industries) supplemented with 10% FBS (GIBCO) in a 5% CO_2_ incubator at 37 °C, respectively.

The lentivirus and miRNA transfection were transfected and established as described previously [41, 42]. The lentiviruses of IGF2BP3, METTL3, and an empty vector were obtained from HanBio (Shanghai, China). The lentiviruses of IGF2BP3-shRNA1/2 and non-target control expressing GFP alone were purchased from GenePharma (Shanghai, China). The shRNA sequences used were as follows: IGF2BP3 shRNA1: 5′-GAAACTTCAGATACGAAATATcgaaATATTTCGTATCTGAAGTTTC-3′; IGF2BP3 shRNA2: 5′-AATCGATGTCCACCGTAAAGAcgaaTCTTTACGGTGGACATCGATT-3′. The scramble negative control (NC), miR320-3p mimic, miR320-3p antagomir, and the riboFECT™ transfection kit were purchased from Ribobio (Guangzhou, China).

### Cell proliferation assays

Cell proliferation was assessed by the CCK-8 and colony formation assays. In the CCK-8 assay, cells were placed into 96-well plates (2000 cells/well) and cultured overnight. The cells were treated with 10 μl CCK-8 (APExBIO) for 1 h at specific time points (0, 24, 48, 72, and 96 h). The OD values at 450 nm were then measured using a microplate reader (BioTek). In colony formation assays, cells were placed in six-well plates (800 cells per well) and incubated for 7 ~ 10 days. The cell colonies were fixed with methanol and stained with crystal violet for 15 min.

### Migration and invasion assays

Cell migration capacities were evaluated via wound-healing assays. The cells were placed in 35-mm plates, and once they reached 90% coverage, a scratch was made using a pipette tip. After being rinsed with PBS, the cells were subsequently incubated in a serum-free medium. The photographs were taken at the specified time intervals to record the scratch closure using a microscope (Olympus IX73).

Cell invasion capacities were evaluated via the transwell assays. Transwell chambers with 8 μm pores (Corning) were pre-coated with Matrigel (BD Biosciences). Cells in serum-free medium were plated to the upper chamber (5 × 10^4^ cells/well) and incubated with medium containing 10% FBS for 24 h. Then, the penetrated cells on the inserts were fixed with 4% paraformaldehyde and stained with Crystal Violet (Beyotime). The cells that invaded to the lower surface of the membrane were photographed with a light microscope.

### Cell cycle and apoptosis analyses

Cell cycle analyses were conducted using Cell Cycle Analysis Kit (Beyotime, C1052, Shanghai, China) as detailed previously [43]. And apoptosis analyses were conducted using Annexin V-AF666/Propidium Iodide (PI) double staining cell apoptosis detection kit (Bestbio, BB-41037, Shanghai, China) as detailed previously [44]. Data were collected by LSRFortessa flow cytometer (BD Biosciences, USA) and analyzed by FlowJo V10 software (Flowjo, LLC).

### RT-qPCR

The RT-qPCR was performed as detailed previously [41, 42]. The primer sequences were as follows (5′ → 3′):

IGF2BP3 forward, TCGTGACCAGACACCTGATGAG;

IGF2BP3 reverse, GGTGCTGCTTTACCTGAGTCAG;

HMGB1 forward, AGATATGGCAAAAGCGGACA;

HMGB1 reverse, GGGCGATACTCAGAGCAGAAG;

GAPDH forward, GGAGCGAGATCCCTCCAAAAT;

GAPDH reverse, GGCTGTTGTCATACTTCTCATGG.

### Bisulfite sequencing PCR (BSP)

The BSP assays in RT112/84 and BFTC905 cells were performed as detailed previously [43]. The primers used for amplifying the upstream CpG island of IGF2BP3 were as follows: forward, 5′-GTGGGGTTAGGTTYGGGTTTA-3′ and reverse, 5′- CAACCACTCTTCACAATAAACAAAC-3′. Sequencing was performed on six different clones of every cell line.

### Demethylating treatment

RT112/84 cells were treated with 30 μM 5AZA-CdR (APExBIO, A1906) for five days, with daily replacement of fresh medium containing 5AZA-CdR. Then, the collected cells were used for subsequent analysis using RT-qPCR and western blotting.

### Luciferase reporter assay

The assay was performed as detailed previously [45]. Briefly, the 3′-UTR of IGF2BP3 containing putative miR-320-3p target region were amplified using the primers: forward 5′-GATGCCAAACCAAAGACAGATT-3′ and reverse 5′-GAAATGTAGCCTTTTGTTGCGT-3′. The product was then cloned to downstream of the firefly luciferase gene in pGL3 luciferase reporter vector, resulting in the construction of pGL3-luc-IGF2BP3 UTR.

### Western blot analysis

The western blots were performed as detailed previously [41, 42]. The primary antibodies utilized in this study were as follows: human IGF2BP3 (Abcam #ab177477), Cyclin B (CST #4138), Cyclin E (Proteintech #11554-1-AP), Snail (Proteintech #13099-1-AP), Slug (Proteintech #12129-1-AP), E-cadherin (Proteintech #20874-1-AP), N-cadherin (Proteintech #22018-1-AP), Vimentin (Proteintech #10366-1-AP), MMP9 (Proteintech #10375-2-AP), METTL3 (Proteintech #15073-1-AP), HMGB1 (Proteintech #10829-1-AP), p65 (CST #8242), phospho-65 (CST #3033), CD44 (Proteintech #15675-1-AP), LC3 (CST, #12741) (P62 (Proteintech #66184-1-Ig), KLF4 (Proteintech #11880-1-AP), and GAPDH (Proteintech #10494-1-AP). The secondary antibodies used in this study were as follows: anti‑rabbit IgG (Proteintech #SA00001-2) or anti‑mouse IgG (ProteinTech #SA00001‑1).

### RBP-target analysis and CLIP-seq analysis of IGF2BP3

ENCORI was utilized to conduct the RBP-target analysis of IGF2BP3. The binding sites between IGF2BP3 and HMGB1 mRNA were presented on the website (https://rnasysu.com/encori/rbpClipRNA.php?source=mRNA&flag=none&clade=mammal&genome=human&assembly=hg38&RBP=IGF2BP3&clipNum=1&regionType=None&pval=0.05&clipType=None&panNum=0&target=HMGB1).

The human IGF2BP3 PAR-CLIP dataset was obtained from GEO (GSE21578, [46]) and analyzed using the POSTAR3 platform (http://111.198.139.65/) [47]. For the CLIP-seq analysis of IGF2BP3 binding to HMGB1 mRNA, the relevant data was searched and obtained from POSTAR3.

### mRNA stability assays

Cells were seeded overnight and then treated with 5 μg/ml actinomycin D (Selleck #RASP-101) to hinder the synthesis of intracellular RNA. At specified time points, total RNA was isolated using TRIzol (Invitrogen #15596026) and quantified by RT-PCR. GraphPad Prism was used to assess mRNA decay kinetics and calculate half-life.

### RNA immunoprecipitation (RIP)

The RIP experiments were performed as detailed previously [48] using an EZ-Magna RIP Kit (Millipore #17–700). Briefly, BFTC905 cell lysates were prepared using lysis buffer and subsequently incubated with magnetic beads conjugated with the specified antibodies (IGF2BP3 and negative control IgG) overnight at 4 °C. The immunocomplexes were digested with proteinase K, followed by RNA extraction and RT-qPCR to detect HMGB1 enrichment.

### Xenograft mouse model for in vivo studies

Ten BALB/c nude mice (~ 6 weeks) were obtained and kept at the Experimental Animal Center of the First Affiliated Hospital of USTC (University of Science and Technology of China). The animal experiments were performed in accordance with the Basel Declaration and were approved by the Ethics Committees of USTC (permission number: 2021-N(A)-297). The mice were provided with pathogen-free cages, supplied with food and water, and maintained under controlled temperature and humidity conditions in a room with a 12-h light/dark cycle. The mice were randomly assigned to two groups, with five in each group. Vector control or IGF2BP3 knockdown BFTC905 cells (1 × 10^6^ cells in 100 μl PBS) were subcutaneously injected into the right flanks of each mouse. From day 6 onwards, the tumor was assessed every 4 days, and volumes were calculated based on the equation: volume = (length × width^2^)/2.

### Statistical analysis

Statistical analysis was undertaken in GraphPad Prism 9 or R software. The experiments in this study were repeated at least three times, and data are presented as means ± standard deviation (SD). Means were compared by Student’s *t* test or Welch’s *t* test. Spearman’s correlation was used in correlation analyses. *p* < 0.05 were considered statistically significant. (^∗^*p* < 0.05; ^∗∗^*p* < 0.01; ^∗∗∗^*p* < 0.001; ^∗∗∗∗^ *p* < 0.0001).

## Results

### IGF2BP3 is highly expressed in various tumors, including bladder cancer

The IGF2BPs family (IGF2BP1/2/3), which are m6A readers, influence the fates of mRNAs in an m6A-dependent manner. Nevertheless, the involvement of IGF2BPs in the advancement of cancer is yet to be investigated, particularly in bladder cancer (BLCA). Analysis of TCGA BLCA dataset revealed that the expression of IGF2BP2 in BLCA was comparable to that in normal tissues (Additional file 1: Fig. S1A), while IGF2BP1 expression did not exhibit a significant association with the overall survival of BLCA patients (Additional file 1: Fig.S1B). Of note, IGF2BP3 was the only gene that is not only overexpressed in bladder cancer, but also its elevated expression was linked to unfavorable overall survival of BLCA patients (Additional file 1: Fig. S1). As a result, we were prompted to explore the possible functions and underlying mechanisms of IGF2BP3 in the advancement of bladder cancer.

**Fig. 1:**
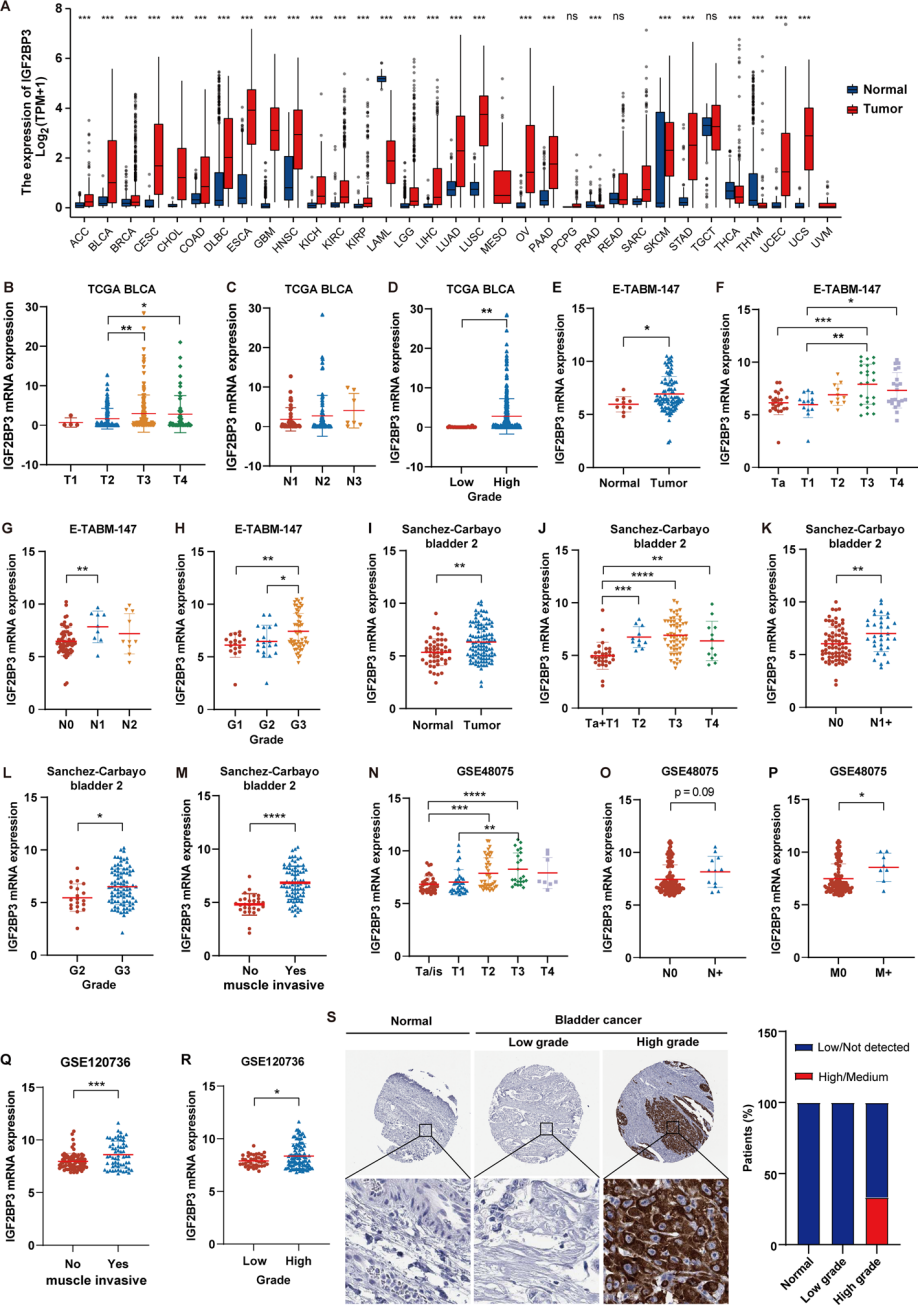
IGF2BP3 expression in normal and cancer tissues. **A** Comparison of IGF2BP3 mRNA levels between tumor and normal tissue across TCGA cancer types by analyzing integrated data from the TCGA and GTEx databases. **B**–**D** IGF2BP3 mRNA levels in bladder cancer samples from the TCGA BLCA dataset, categorized by T stages, N stages, and histologic grade. **E**–**H** IGF2BP3 mRNA levels in bladder cancer samples from the E-TABM-147 dataset, categorized by tissue type (normal vs. cancer), T stages, N stages, and histologic grade. **I**–**M** IGF2BP3 mRNA levels in bladder cancer samples from the “Sanchez-Carbayo bladder 2” dataset, categorized by tissue type, T stages, N stages, histologic grade, and muscle-invasive type. **N**–**P** IGF2BP3 mRNA levels in bladder cancer samples from the GSE48075 dataset, categorized by T stages, N stages, and M stages. **Q**–**R** IGF2BP3 mRNA levels in bladder cancer samples from the GSE120736 dataset, categorized by muscle invasive type and histologic grade. **S** Representative immunohistochemical images of IGF2BP3 in normal bladder tissue, low-grade and high-grade bladder cancer tissues in Human Protein Atlas (HPA). The percentage of different staining intensities in each type of tissue is shown in a stacked bar graph. **** *p* < 0.0001, *** *p* < 0.001, ** *p* < 0.01, * *p* < 0.05

To assess the expression profiles of IGF2BP3, we analyzed its expression levels in 33 different types of tumor and normal tissues through integrated data from TCGA and GTEx databases. The expression of IGF2BP3 was markedly elevated in the majority of examined tumors, such as BLCA, in comparison to their respective normal tissues (*p* < 0.001, Fig. 1A, Additional file 1: Fig. S1A). To further assess the expression of IGF2BP3 in human BLCA, we analyzed the mRNA levels of IGF2BP3 in two additional BLCA datasets, including E-TABM-147 and “Sanchez-Carbayo bladder 2” dataset. Consistently, we observed a notable elevation in IGF2BP3 transcript levels in bladder cancer tissues compared to normal tissues (Fig. 1E, I).

We further assessed the correlation between IGF2BP3 expression levels and the clinicopathological features of bladder cancer using multiple datasets. Our analysis revealed that high IGF2BP3 expression was linked to more advanced T stage (Fig. 1B, F, J, N, Additional file 2: Fig. S2B, D, F, H, J, M, and P). This indicated a potential role of IGF2BP3 in bladder cancer proliferation. Additionally, increased IGF2BP3 expression were linked to more advanced N stage (Fig. 1C, G, K, and O) and M stage (Fig. 1P), as well as higher grade BLCA (Fig. 1D, H, L, R, Additional file 2: Fig. S2C, E, G, K, and N). And its expression was higher in infiltrating bladder cancer than superficial bladder cancer (Fig. 1M, Q, Additional file 2: Fig. S2I, L, and O). These findings indicated its participation in the invasion and spread of bladder cancer.

Furthermore, we analyzed the protein expression of IGF2BP3 using the Human Protein Atlas (HPA) database. IGF2BP3 protein was mainly localized in the cell cytoplasm. While it exhibited low expression in normal urothelial bladder samples, around 34% of high-grade bladder cancer samples showed high levels of IGF2BP3 protein (Fig. 1S), consistent with the mRNA data.

Collectively, our results suggest that IGF2BP3 is upregulated in bladder carcinoma, and its elevated expression is linked to unfavorable clinicopathological characteristics, implying its possible oncogenic function in bladder cancer.

### IGF2BP3 acts as a putative prognostic factor in bladder cancer

**Fig. 2:**
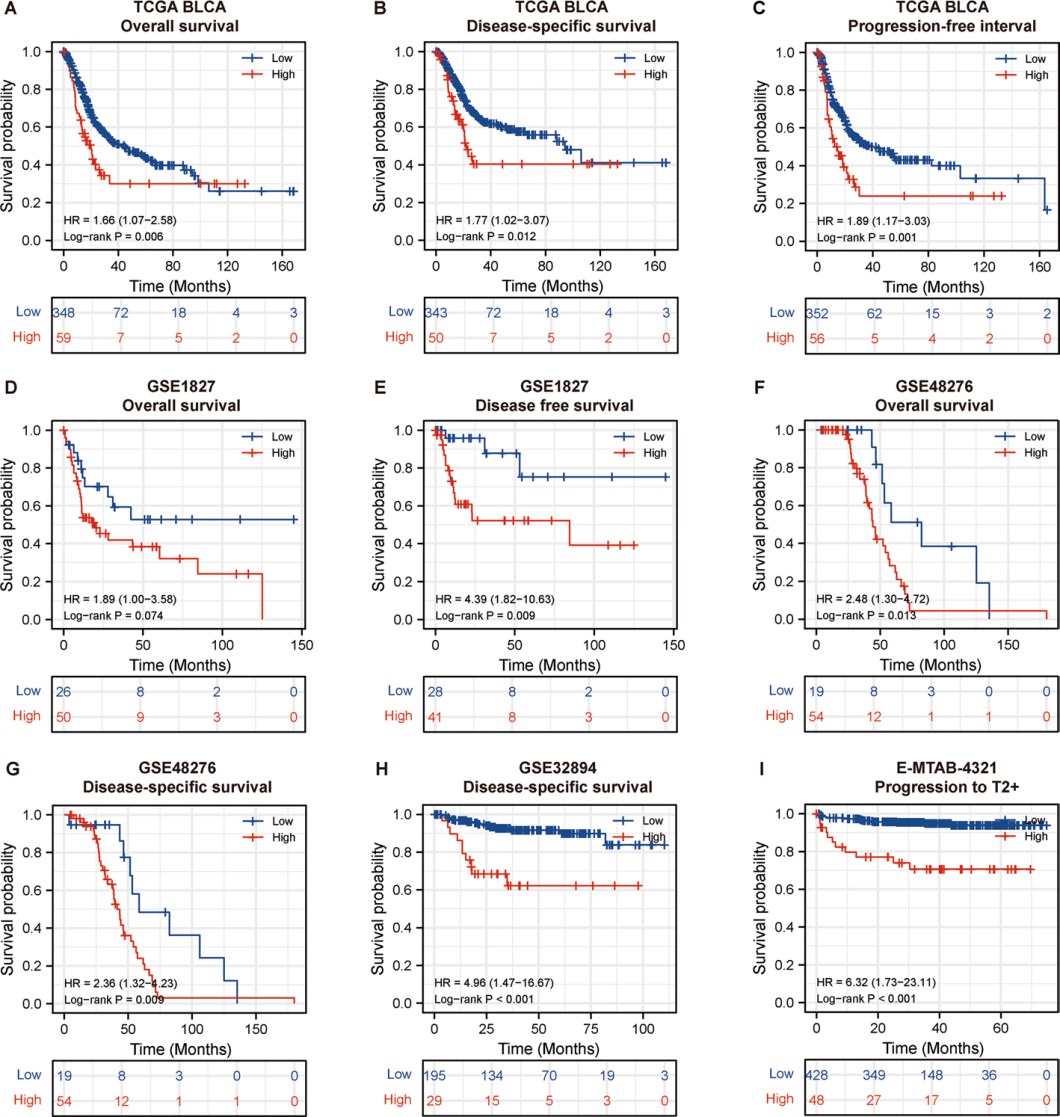
High IGF2BP3 expression correlated with unfavorable outcomes in bladder cancer patients. **A**–**C** Kaplan–Meier analysis of OS, DSS, and PFI according to IGF2BP3 expression in TCGA BLCA. **D**, **E** Kaplan–Meier analysis of OS and DFS according to IGF2BP3 expression in GSE1827. **F**, **G** Kaplan–Meier analysis of OS and DSS according to IGF2BP3 expression in GSE48276. **H** Kaplan–Meier analysis of DSS according to IGF2BP3 expression in GSE32894. **I** Kaplan–Meier analysis of PFS for patients with early-stage bladder cancer according to IGF2BP3 expression in E-MTAB-4321. OS, overall survival; DSS, disease specific survival, progression free interval (PFI), disease free survival (DFS), progression-free survival (PFS)

The prognostic significance of IGF2BP3 expression in BLCA was further investigated. The analysis using Kaplan–Meier Plotter revealed a significant correlation between elevated IGF2BP3 levels and reduced overall survival (OS) (*p =* 0.006), disease-specific survival (DSS) (*p =* 0.012), and progression-free interval (PFI) (*p =* 0.001) in the TCGA BLCA dataset (Fig. 2A–C, Additional file 2: Fig.S2B). In addition, the association between elevated IGF2BP3 levels and unfavorable survival was validated in six additional bladder cancer datasets (Fig. 2D–I). Elevated levels of IGF2BP3 were linked to decreased overall survival in GSE1827 (*p =* 0.074, Fig. 2D) and GSE48276 (*p =* 0.013, Fig. 2F) datasets. Furthermore, increased IGF2BP3 levels were linked to poorer disease-free survival (DFS) in GSE1827 (*p =* 0.009, Fig. 2E) and DSS in GSE48276 (*p =* 0.009, Fig. 2G), GSE32894 (p < 0.001, Fig. 2H) datasets. Examination of E-MTAB-4321 dataset, which consisted of 476 early-stage bladder cancer, revealed that tumors exhibiting elevated IGF2BP3 levels had a higher tendency to progress to advanced T stage (*p* < 0.001, Fig. 2I).

These findings collectively indicate that IGF2BP3 expression can serve as a prognostic predictor for bladder cancer patients, with elevated levels of expression linked to worse clinical outcomes.

### Triple mechanisms of IGF2BP3 gene expression regulation

**Fig. 3:**
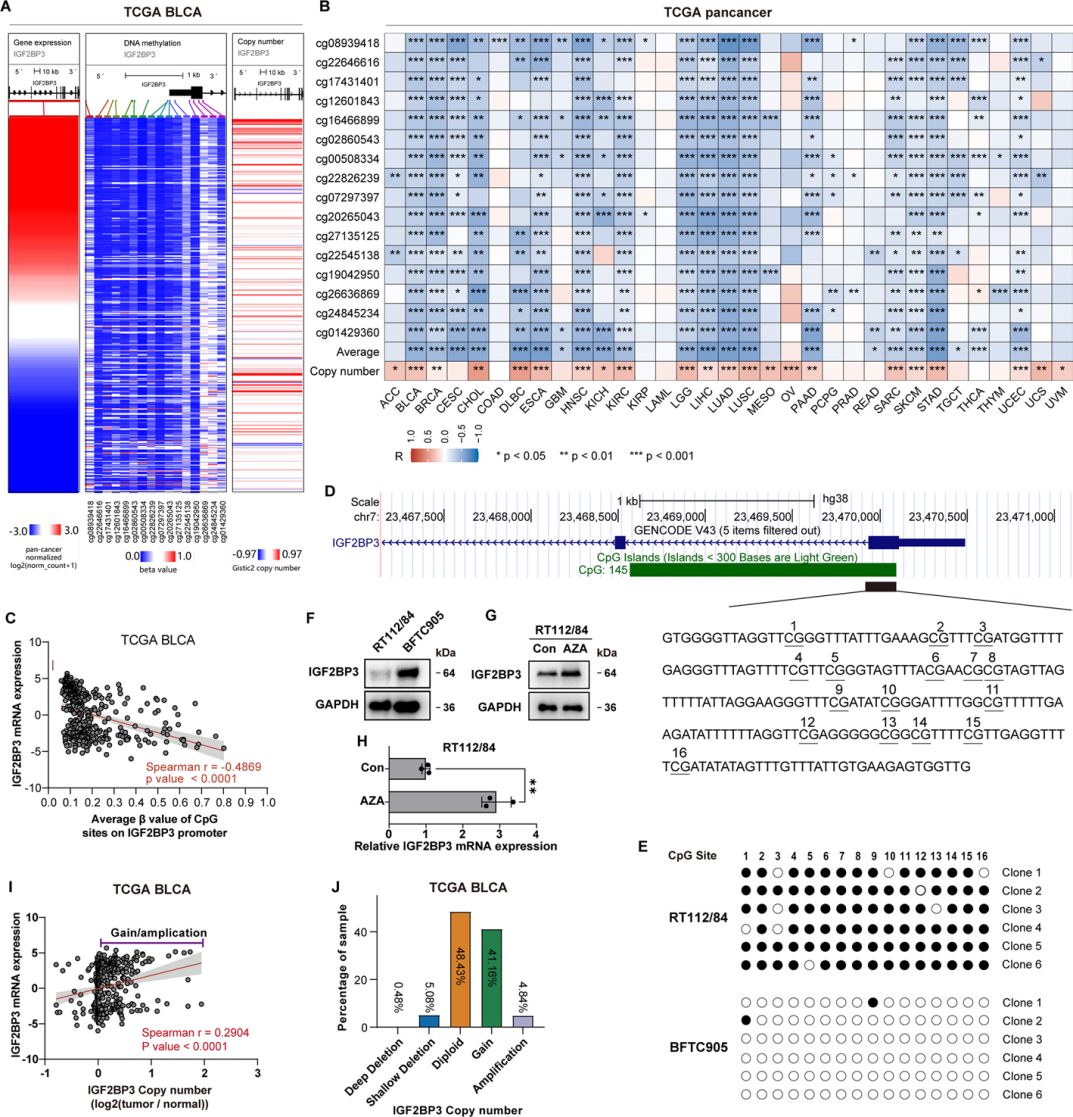
High IGF2BP3 expression is induced by promoter hypomethylation and copy number gain/amplification in various tumors, including bladder cancer. **A** Heatmap representing IGF2BP3 mRNA levels and β-value (methylation level) of 16 CpG sites on the IGF2BP3 promoter. Sorted by IGF2BP3 mRNA levels. Blue: low value, red: high value. **B** Heatmap displaying the Spearman correlation coefficient between IGF2BP3 mRNA levels and β-value of CpG sites on its promoter, as well as copy number of the gene across 33 cancer types in TCGA. **C** Scatterplot showing the positive correlation between IGF2BP3 mRNA level and the average β-value of CpG sites on the IGF2BP3 promoter in TCGA BLCA. **D** The UCSC Genome Browser displays the IGF2BP3 gene along with its upstream region. Within the CpG Island of the IGF2BP3 promoter region, 16 CpG sites are underlined. These CpG sites were examined using BSP (bisulfite sequencing PCR). **E** Methylation status of the CpG sites presented in **D** in RT112/84 and BFTC905 cells. Methylated CpG sites are depicted as solid circles, whereas unmethylated CpG sites are illustrated as open circles. **F** WB analysis of IGF2BP3 protein levels in RT112/84 and BFTC905 cells. **G**, **H** The impact of 5-aza treatment on the protein and mRNA levels of IGF2BP3 in RT112/84 cells. **I** Correlation analysis of IGF2BP3 expression and copy number in TCGA BLCA. **J** The frequency of samples exhibiting distinct copy number alterations of IGF2BP3 in TCGA BLCA. Error bars indicate the mean ± sd from three independent experiments. ***p < 0.001, **p < 0.01, *p < 0.05

Subsequently, we delved deeper into the mechanism behind the upregulation of IGF2BP3 in bladder cancer. DNA hypomethylation is a prevailing mechanism responsible for the deregulation of oncogenes in tumors [49]. We focused our analysis on the methylation alterations of 16 CpG sites located on the IGF2BP3 gene promoter, using the DNA methylation array data from TCGA. The analysis showed that the methylation levels of the majority of the CpG sites we examined showed a strong negative correlation with the IGF2BP3 mRNA levels in various tumor types from the TCGA datasets, including TCGA BLCA (Fig. 3A, B). Also, the average methylation level of these sites negatively correlated with IGF2BP3 mRNA levels in most of the tumor types in TCGA datasets (Fig. 3B). Specifically, in TCGA BLCA, both the methylation levels of 16 CpG sites and the average methylation level showed a strong and significant negative correlation with IGF2BP3 mRNA levels (Fig. 3A–C). To further substantiate these bioinformatics findings, we examined the levels of expression and methylation in the promoter region of IGF2BP3 in BLCA cells, specifically RT112/84 and BFTC905 cells. Bisulfite sequencing was employed to investigate the methylation levels of specific regions on the IGF2BP3 promoter (Fig. 3D). The CpG sites in the IGF2BP3 promoter of BFTC905 exhibited reduced methylation levels compared to RT112/84, whereas the protein level was higher in BFTC905 than RT112/84 (Fig. 3F). Furthermore, blocking methylation with 5-aza considerably increased the protein and mRNA levels of IGF2BP3 in RT112/84 cells (Fig. 3G, H). These findings suggest a significant contribution of promoter methylation to the regulation of IGF2BP3 expression.

Gene expression is also closely linked to DNA copy number alteration (CNA). Therefore, we next integrated the copy number and mRNA expression data of IGF2BP3 from the TCGA dataset for joint analysis to examine whether CNA affects the mRNA expression of IGF2BP3. Upon analyzing this data, it was discovered that the IGF2BP3 copy number was often amplified in bladder cancer of TCGA BLCA dataset (Fig. 3I, J). Moreover, IGF2BP3 copy number positively correlated with its mRNA levels (*r* = 0.2904, *p* < 0.0001; Fig. 3A, B, I). Furthermore, we noticed a significant positive association between IGF2BP3 mRNA levels and its copy number across 23 different tumor types in TCGA datasets (Fig. 3B). These findings suggest that copy number gain/amplification of IGF2BP3 contributes to its overexpression in most tumor types, including bladder cancer.

**Fig. 4:**
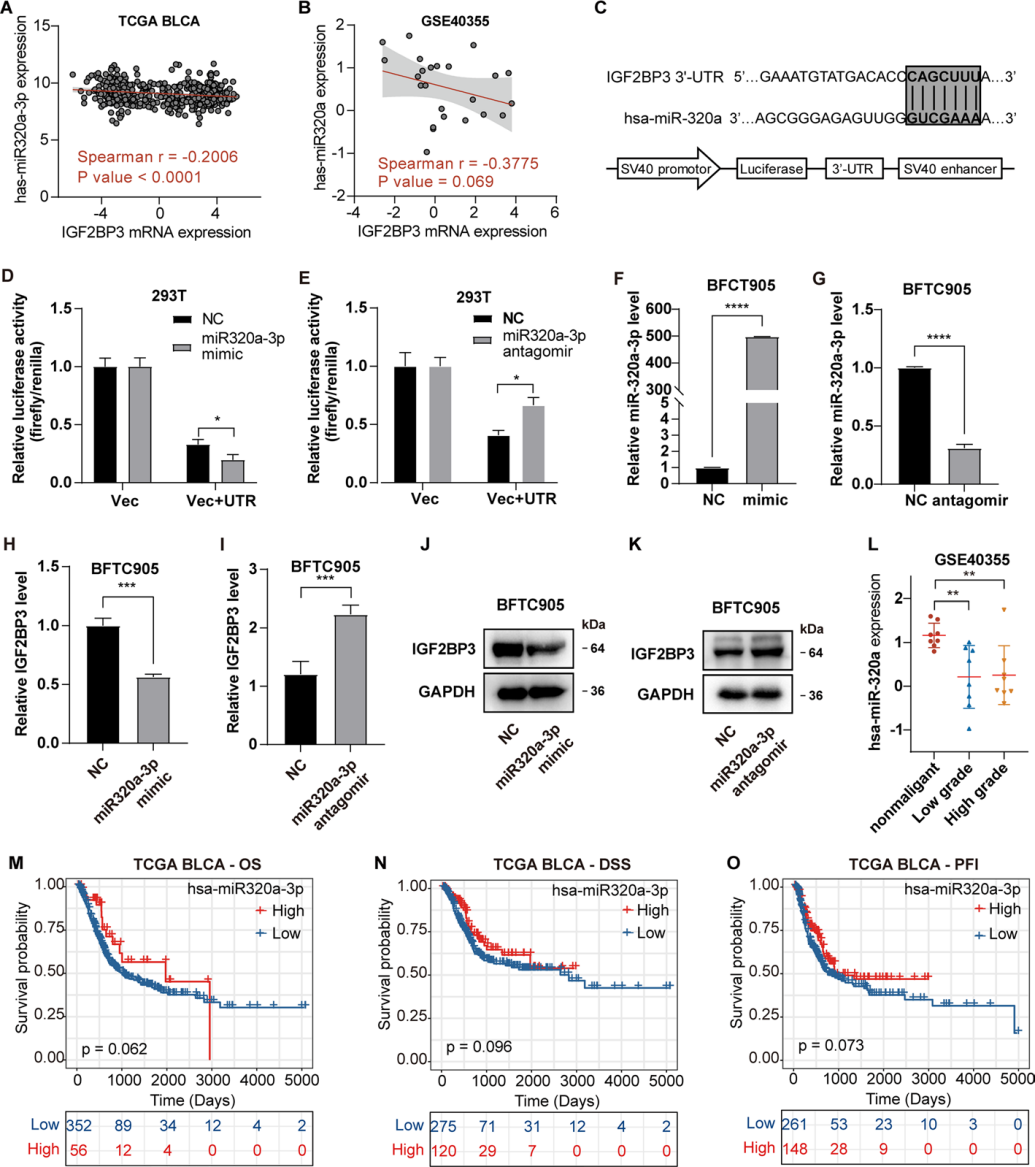
IGF2BP3 expression is negatively regulated by miR-320a-3p in bladder cancer. **A** Correlation analysis between IGF2BP3 mRNA level and miR-320a-3p level in TCGA BLCA. **B** Correlation analysis between IGF2BP3 mRNA level and miR-320a level in GSE40355. **C** Construction of a reporter plasmid with the 3′-UTR of the IGF2BP3 gene targeted by miR-320a-3p. **D**, **E** Determination of the relative luciferase activity in 293 T cells transfected with the miR-320a-3p mimic, antagomir, or negative control (NC). Vec, vector without UTR sequence. **F**, **G** The relative miR-320a-3p level in BFTC905 cells after treatment with miR-320a-3p mimic or antagomir. **H**–**K** The effect of miR-320a-3p mimic, antagomir on the IGF2BP3 mRNA and protein level in BFTC905 cells. **L** Comparison of miR-320a-3p levels between normal and bladder cancer tissues in GSE40355 dataset. **M**–**O** Kaplan–Meier analysis of OS, DSS, and PFI according to miR-320a-3p expression in the TCGA BLCA. Error bars indicate the mean ± sd from three independent experiments. ***p < 0.001, **p < 0.01, *p < 0.05

It's widely recognized that microRNAs (miRNAs) can repress gene expression through binding to 3′-UTR of the targeted mRNA. Utilizing the miRDB prediction tool (http://mirdb.org/miRDB/), we identified 260 miRNAs predicted to target IGF2BP3. Out of these miRNAs, miR-320a-3p displayed a relatively high target score (score = 89), and its level significantly negatively correlated with IGF2BP3 levels in TCGA BLCA (*r* =  − 0.2006, *p* < 0.0001, Fig. 4A). This negative correlation was also validated using the bladder cancer dataset GSE40355 (Fig. 4B). In order to confirm the direct binding of miR-320a-3p and IGF2BP3, we inserted the 3′-UTR of IGF2BP3 into the downstream of the firefly luciferase gene in pGL3-control vector, resulting in the formation of the pGL3-IGF2BP3-UTR construct (Fig. 4C). Upon transfecting in 293 T cells, it was observed that the luciferase activity of pGL3-IGF2BP3-UTR was significantly reduced in cells transfected with the miR-320a-3p mimic, while significantly increased in cells transfected with miR-320a-3p antagomir (Fig. 4D, E). Furthermore, miR-320a-3p mimic transfection also resulted in a significant reduction of IGF2BP3 both at the mRNA and protein levels in BFTC905 cells, while transfection with miR-320a-3p antagomir resulted in the opposite effect (Fig. 4F–K). Of note, we revealed that miR-320a-3p expression in bladder cancer was significantly lower than in para-carcinoma tissues by analyzing GSE40355 dataset (Fig. 4L). Kaplan–Meier analysis showed that low miR-320a-3p level was associated with reduced OS (*p =* 0.062), DSS (*p =* 0.096), and PFI (*p =* 0.073) in TCGA BLCA (Fig. 4M–O). This indicates a poor prognosis for BLCA patients expressing low levels of miR-320a-3p.

In conclusion, our results demonstrate that IGF2BP3 is negatively regulated by promoter methylation and miR-320a-3p, but positively regulated by CNA in bladder cancer. Additionally, this methylation and CNA induced regulation of IGF2BP3 expression are likely to be prevalent in most tumors.

### IGF2BP3 promotes bladder cancer progression

To determine the pathways associated with IGF2BP3 expression in bladder cancer, we utilized Gene Set Enrichment Analysis (GSEA) to identify gene sets that exhibited enrichment or depletion in tumors with high IGF2BP3 expression. This analysis was performed on three BLCA datasets: TCGA BLCA, GSE31684, and E-MTAB-4321, each containing over 90 BLCA samples. We focused on the common gene sets associated with IGF2BP3 expression across all three datasets (Additional file 5: Table S1).

**Fig. 5:**
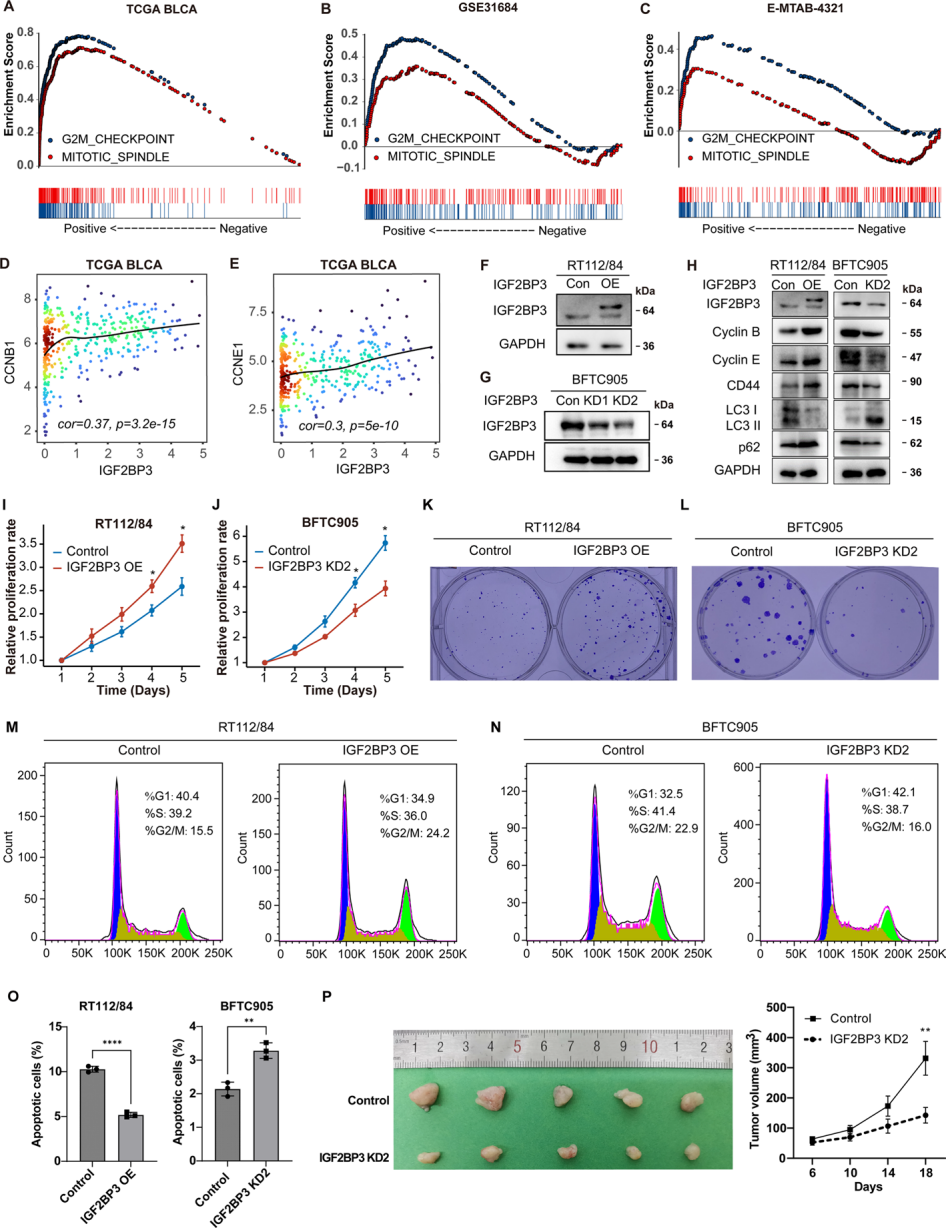
IGF2BP3 promotes the proliferation of bladder cancer cells. **A**–**C** GSEA analysis demonstrates a positive correlation between IGF2BP3 mRNA levels and HALLMARKs of "G2M CHECKPOINT" and "MITOTIC SPINDLE" signatures in the TCGA BLCA, GSE31684, and E-MTAB-4321 datasets. **D**, **E** Scatterplot showing the mRNA expression correlation between IGF2BP3 and CCNB1/CCNE1 in the TCGA BLCA dataset. **F**, **G** Western blot analysis confirming the efficacy of IGF2BP3 overexpression in RT112/84 cells, and silencing by two independent shRNAs in BFTC905 cells. **H** Western blot analysis of Cyclin B, Cyclin E, CD44, LC-3, and p62 protein levels after IGF2BP3 overexpression in RT112/84 cells and IGF2BP3 knockdown in BFTC905 cells. **I**, **J** The effect of IGF2BP3 expression on cell proliferation in RT112/84 cells and IGF2BP3 knockdown in BFTC905 cells was assessed by CCK8 assay. **K**, **L** Representative images of colony formation assays in RT112/84 vs. IGF2BP3-OE RT112/84 cells, and BFTC905 vs. IGF2BP3-KD BFTC905 cells, respectively. **M**, **N** Flow cytometric analysis of the effect of IGF2BP3 expression on cell cycle distribution in RT112/84 and BFTC905 cells. **O** Proportions of apoptotic cells. **P** IGF2BP3 knockdown in BFTC905 inhibited tumor growth in xenograft mice model. The tumor diameter was measured, and tumor volume was calculated every 4 days. Con control, OE overexpression, KD knockdown. **p < 0.01, *p < 0.05

In all three BLCA datasets, we discovered that the activity of cell cycle associated HALLMARKs, including “MITOTIC SPINDLE” and “G2M CHECKPOINT”, was significantly positively associated with IGF2BP3 mRNA levels. Furthermore, the mRNA levels of cyclin B1 (CCNB1) and cyclin E1 (CCNE1), which are markers of mitosis, exhibited a positive correlation with IGF2BP3 expression in TCGA BLCA (*r* = 0.37, *r* = 0.3, respectively) (Fig. 5D, E). Combined with the results that high IGF2BP3 levels correlate with higher T stage (Fig. 1), these findings suggest that bladder cancer with high IGF2BP3 levels exhibits fast cell proliferation.

To examine the potential oncogenic function of IGF2BP3 in the development of bladder cancer, we conducted overexpression and loss-of-function studies. We stably transfected the bladder cancer cell lines RT112/84 with an IGF2BP3 expression lentivirus or a control lentivirus (Fig. 5F). To suppress IGF2BP3 expression in BFTC905 cells, we designed two small interference RNAs (KD1 and KD2). Transfection with these siRNAs effectively reduced the expression of IGF2BP3, particularly KD2 (Fig. 5G). Furthermore, IGF2BP3 overexpression increased the levels of cyclin B and cyclin E proteins, while IGF2BP3 knockdown decreased their expression levels (Fig. 5H). Functionally, CCK-8 assays revealed that IGF2BP3 overexpression promoted the growth of RT112/84 cells, whereas IGF2BP3 knockdown significantly impeded the proliferation of BFTC905 cells (Fig. 5I, J). Additionally, colony-forming assays provided further confirmation of the enhancing impact of IGF2BP3 on the growth of bladder cancer cells (Fig. 5K, L). To elucidate the underlying mechanism, we first performed cell cycle analyses. Overexpression of IGF2BP3 in RT112/84 cells increased cells in S + G2/M phases, whereas knocking down IGF2BP3 in BFTC905 cells resulted in a decrease in the percentage of cells in the S + G2/M phases (Fig. 5M, N). We also observed that IGF2BP3 could enhance the expression of CD44 (Fig. 5H), which is a well-known biomarker of cancer stem cells that are typically associated with increased proliferation capacity [50]. Additionally, we discovered that IGF2BP3 could significantly inhibit autophagy, as indicated by the accumulation of p62 and reduced levels of LC3-II (Fig. 5H). This suggests that IGF2BP3 is likely to suppress autophagic cell death in bladder cancer cells. And, flow cytometry analysis for apoptosis demonstrated that overexpression of IGF2BP3 led to a significant decrease in the percentage of apoptotic cells, whereas knocking down IGF2BP3 increased the percentage of apoptotic cells (Fig. 5O and Additional file 3: Fig.S3). To investigate the role of IGF2BP3 in promoting the progression of bladder cancer in vivo, we created xenograft models in BALB/c nude mice by implanting BFTC905 cells (control vs. IGF2BP3 knockdown). As shown in 5P, IGF2BP3 knockdown significantly inhibited bladder carcinoma tumor growth.

**Fig. 6:**
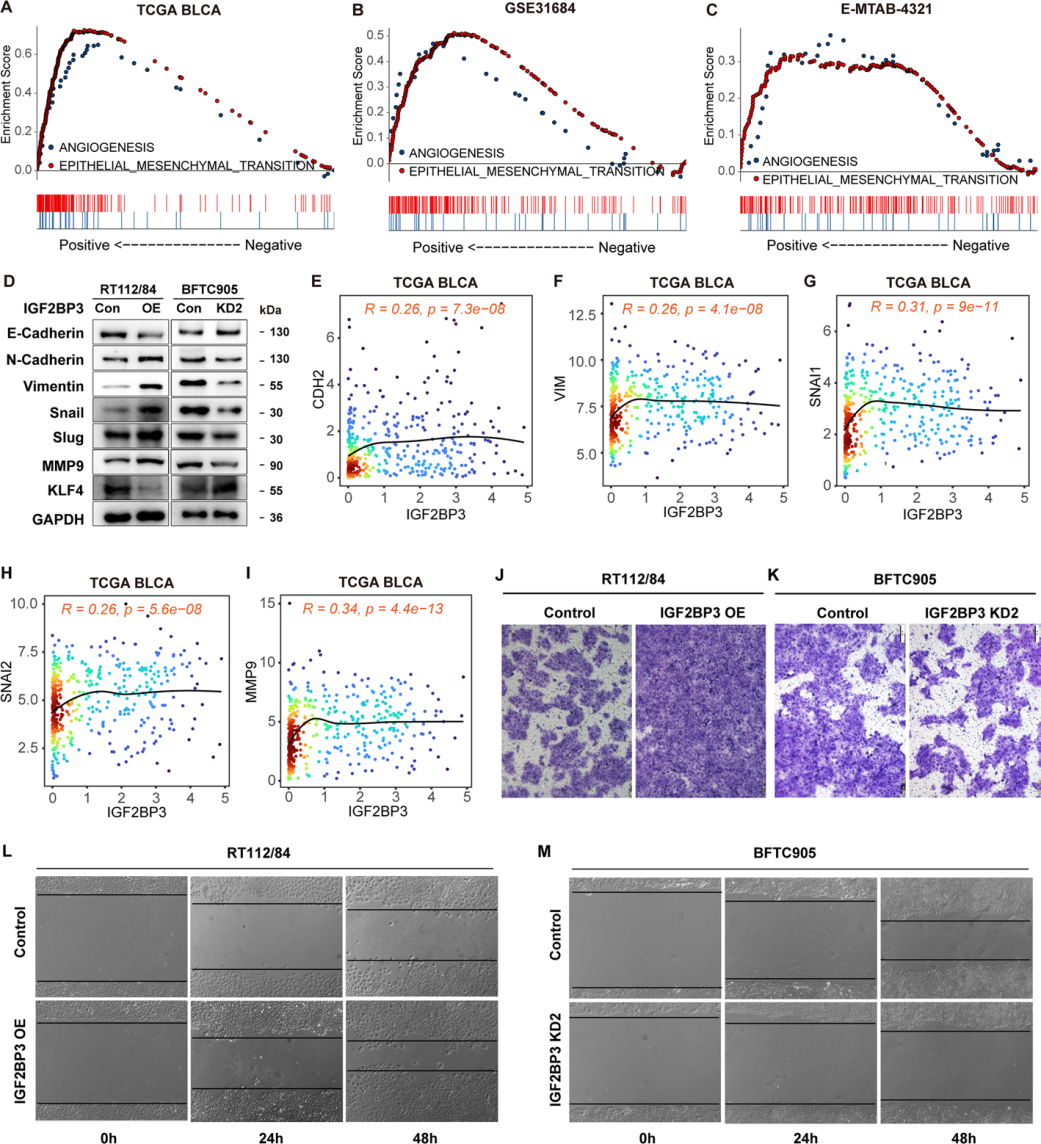
IGF2BP3 promotes EMT, migration, invasion, and metastasis of bladder cancer cells. **A**–**C** GSEA analysis reveals a positive correlation between IGF2BP3 mRNA levels and HALLMARKs of “ANGIOGENESIS” and “EPITHELIAL MESENCHYMAL TRANSITION” signature in TCGA BLCA, GSE31684 and E-MTAB-4321 datasets. **D** Western blot analysis of E-cadherin, N-cadherin, Vimentin, Snail, Slug, MMP9, and KLF4 protein levels after IGF2BP3 overexpression in RT112/84 and IGF2BP3 knockdown in BFTC905 cells. **E**–**I** Scatterplot exhibiting the mRNA expression correlation between IGF2BP3 and CDH2/VIM/SNAI1/SNAI2/MMP9 in the TCGA BLCA dataset. **J**, **K** Assessment of the effect of IGF2BP3 expression on cell invasion in RT112/84 and BFTC905 cells using a transwell assay. **L**, **M** Evaluation of the effect of IGF2BP3 expression on cell migration in RT112/84 and BFTC905 cells through a wound-healing assay

High levels of IGF2BP3 were found to be correlated with higher N/M stages and invasion in bladder cancer (Fig. 1). Additionally, GSEA analysis identified a positive correlation between IGF2BP3 levels in BLCA and gene sets related to "ANGIOGENESIS" and "EPITHELIAL MESENCHYMAL TRANSITION" (EMT), both of which are associated with cancer metastasis [51–53]. This association was observed across the TCGA BLCA, GSE31684, and E-MTAB-4321 datasets (Fig. 6A–C). Further examination of the influence of IGF2BP3 on EMT markers showed that in IGF2BP3-overexpressing cells, the level of E-cadherin, an epithelial marker, was found reduced, whereas the levels of mesenchymal markers such as vimentin and N-cadherin increased compared to vector-expressing RT112/84 cells (Fig. 6D). Conversely, E-cadherin was upregulated in BFTC905 cells with IGF2BP3-silencing, whereas N-cadherin and vimentin were downregulated. Additionally, screening of EMT-related transcription factors revealed that IGF2BP3 significantly induced the protein expression of Snail and Slug. These findings indicate that IGF2BP3 promotes EMT in BLCA cells. Moreover, it was observed that IGF2BP3 increased the expression of MMP9, which stimulates tumor invasion and metastasis via the degradation of the extracellular matrix and is also important for EMT [54]. Correlation analysis in bladder cancer samples from TCGA BLCA demonstrated a positive correlation between mRNA levels of IGF2BP3 and SNAI1 (Snail), SNAI2 (Slug), CDH2 (N-cadherin), VIM (vimentin), and MMP9 (Fig. 6E–I), further supporting the association between IGF2BP3 and EMT-related markers. Functionally, transwell and wound healing assays indicated that IGF2BP3 overexpression enhanced the invasion and migration capacity of RT112/84 cells, while IGF2BP3 silencing weakened the invasion and migration capacity of BFTC905 cells (Fig. 6J–M). Of note, we found that IGF2BP3 could significantly inhibit the expression of KLF4 (Fig. 6D), which is a major repressor of EMT and key driver of differentiation [55]. This suggests that IGF2BP3 may not only promote EMT by inhibiting KLF4, but also prevent bladder cancer cell differentiation.

Overall, our findings support the notion that IGF2BP3 promotes the progression of bladder cancer by affecting cell proliferation, EMT phenotype, and invasive behavior.

### IGF2BP3 directly interacts with HMGB1 mRNA and increases its stability

**Fig. 7:**
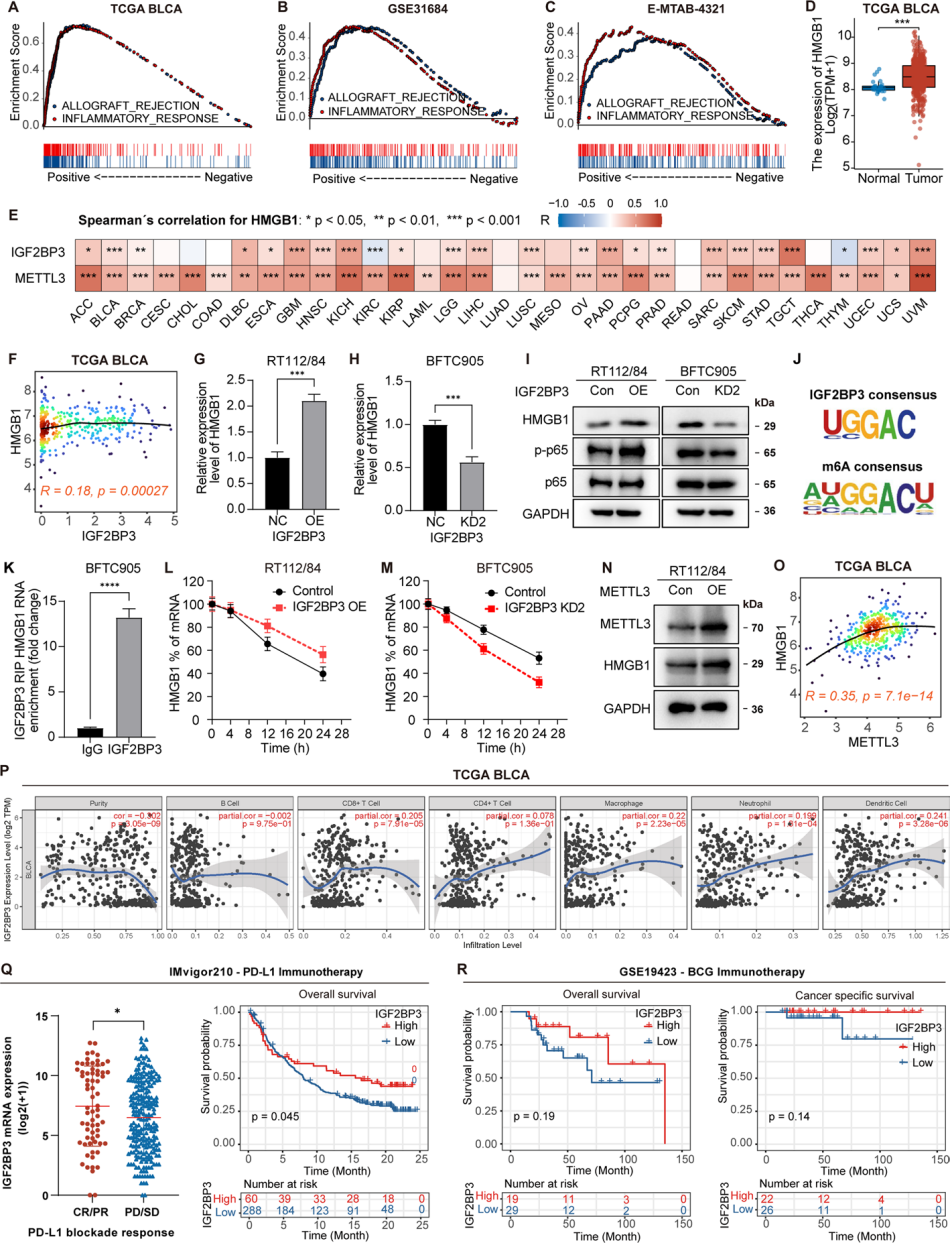
IGF2BP3 promotes the stability of HMGB1 mRNA and its expression in an m6A-dependent manner. **A**–**C** GSEA analysis reveals a positive correlation between IGF2BP3 mRNA levels and HALLMARKs of “INFLAMMATORY RESPONSE” and “ALLOGRAFT REJECTION” signatures in the TCGA BLCA, GSE31684, and E-MTAB-4321 datasets. **D** Comparison of HMGB1 mRNA levels between tumor and normal tissue in the TCGA BLCA dataset. **E** Heatmap showing the Spearman correlation coefficient between HMGB1 mRNA levels and IGF2BP3/METTL3 across 33 cancer types in the TCGA. **F** Scatterplot showing the mRNA expression correlation between IGF2BP3 and HMGB1 in the TCGA BLCA dataset. **G**, **H** RT-qPCR analysis of HMGB1 mRNA levels after IGF2BP3 overexpression in RT112/84 cells and IGF2BP3 knockdown in BFTC905 cells. **I** Western blot analysis of HMGB1, p65, and p-p65 protein levels after IGF2BP3 overexpression in RT112/84 cells and IGF2BP3 knockdown in BFTC905 cells. **J** Motif display of IGF2BP3 targeting consensus and the motif of m6A site. **K** RIP assays followed by RT-qPCR showing the binding of HMGB1 mRNA with IGF2BP3 protein. **L**, **M** RT-qPCR analysis of HMGB1 mRNA at different time points after treatment with actinomycin D (5 μg/ml) in RT112/84 cells following IGF2BP3 overexpression and in BFTC905 cells following IGF2BP3 knockdown. **N** Western blot analysis of HMGB1 protein levels after METTL3 overexpression in RT112/84 cells. **O** Scatterplot showing the mRNA expression correlation between METTL3 and HMGB1 in the TCGA BLCA dataset. **P** Correlation analysis of IGF2BP3 levels and tumor-infiltrating immune cells using the TIMER webtool. **Q** IGF2BP3 expression in different response groups; Kaplan–Meier overall survival (OS) estimates according to IGF2BP3 expression in bladder cancer patients treated with anti-PD-L1 antibody (atezolizumab) from “IMvigor210” dataset. **R** Kaplan–Meier OS and cancer-specific survival (CSS) estimates according to IGF2BP3 expression in the GSE19423 dataset of bladder cancer patients treated with Bacillus Calmette-Guérin (BCG) Immunotherapy. Kaplan–Meier survival curves with *p*-values derived from the log-rank test are shown. **** *p* < 0.0001, *** *p* < 0.001, ** *p* < 0.01, * *p* < 0.05

We next explored the underlying molecular mechanisms of IGF2BP3 in the advancement of bladder cancer. GSEA analysis indicated a positive correlation between IGF2BP3 levels and “INFLAMMATORY RESPONSE” and “ALLOGRAFT REJECTION” in bladder cancer datasets (Fig. 7A–C). Given the involvement of HMGB1 in the inflammatory response, tumorigenesis, and allograft rejection [56–58], and its contribution to the progression of bladder cancer [59–62], we investigated the relationship between IGF2BP3 and HMGB1.

Analysis of TCGA BLCA dataset revealed that mRNA levels of HMGB1 were elevated in bladder cancer samples as compared to normal tissues (Fig. 7D). Additionally, there was a positive correlation observed between HMGB1 levels and IGF2BP3 levels (Fig. 7F). Notably, this significant positive correlation was also found in 22 tumors by analysis of the other 32 tumor datasets in the TCGA (Fig. 7E). Notably, this significant positive correlation was also found in 22 tumors by analysis of the other 32 tumor datasets in the TCGA. We therefore hypothesized that IGF2BP3 could elevate HMGB1 expression and promote tumor progression. Then, by RT-qPCR and western blotting, we found that IGF2BP3 induced both mRNA and protein expression of HMGB1 in RT112/84 cells (Fig. 7G, I). Additionally, the phosphorylation of p65, which could be activated by HMGB1, was enhanced by IGF2BP3 overexpression, whereas the expression of p65 remained unchanged (Fig. 7I). In contrast, silencing of IGF2BP3 in BFTC905 cells led to a decrease in HMGB1 expression and phosphorylation of p65 (Fig. 7H, I). These findings further support the regulatory role of IGF2BP3 in HMGB1 expression and downstream signaling in bladder cancer.

It is known that the IGF2BP3 protein recognizes a specific m6A consensus motif to stabilize m6A-modified RNA (Fig. 7J) [63], leading to increased expression of the target mRNA. In the case of HMGB1 mRNA, there are multiple m6A motifs that can potentially bind to IGF2BP3. RBP-Target analysis using the ENCORI platform presented 35 supported binding sites between IGF2BP3 and HMGB1 mRNA (https://rnasysu.com/encori/rbpClipRNA.php?source=mRNA&flag=none&clade=mammal&genome=human&assembly=hg38&RBP=IGF2BP3&clipNum=1&regionType=None&pval=0.05&clipType=None&panNum=0&target=HMGB1), and analysis of PAR-CLIP data in GSE21578 through POSTAR3 also presented 28 binding sites between IGF2BP3 and HMGB1 mRNA (Additional file 6: Table S2). Moreover, RNA immunoprecipitation (RIP) assays with IGF2PB3 antibody showed that HMGB1 mRNA was enriched with anti-IGF2BP3 antibody relative to the nonspecific IgG control (Fig. 7K), confirming the interaction between IGF2BP3 and HMGB1 mRNA. Additionally, RNA stability assays demonstrated that overexpression of IGF2BP3 decreased the decay rate of HMGB1 mRNA (half-life: 18 h for RT112/84 control cells and 30 h for RT112/84 IGF2BP3 OE cells), while knockdown of IGF2BP3 increased its decay rate (half-life: 27 h for BFTC905 control cells and 15 h for BFTC905 IGF2BP3 KD2 cells) (Fig. 7L, M). These findings suggest that IGF2BP3 regulates the stability of HMGB1 mRNA. Furthermore, we observed that overexpression of METTL3, an m6A writer enzyme, drastically enhanced the expression of HMGB1 (Fig. 7I), suggesting a potential relationship between m6A modification and HMGB1 expression. Correlation analysis on 33 tumor types from the TCGA database revealed a strong positive correlation between METTL3 and HMGB1 expression in 31 tumor types, including BLCA (Fig. 7E, O). These observations support the hypothesis that the interaction between IGF2BP3 and HMGB1 mRNA, possibly mediated by m6A modifications, contributes to the regulation of HMGB1 expression in various tumors, including bladder cancer.

Given the association of HMGB1 with the inflammatory response and immune microenvironment, our study aimed to explore the relationship between the IGF2BP3 level and the immune response in bladder cancer. First, we observed that IGF2BP3 level positively correlated with the infiltration level of various immune cells, including CD8 + T cell, macrophage, neutrophil, and dendritic cell in TCGA BLCA dataset, through analysis using a web-tool TIMER (https://cistrome.shinyapps.io/timer/) (Fig. 7P). Given that these cells, especially the CD8 + T cells, play important roles in immunotherapy, we investigated whether IGF2BP3 level was associated with immunotherapy efficacy in bladder cancer. Analysis of the IMvigor210, a large phase 2 trial that investigated efficacy and safety of anti-PD-L1 antibody atezolizumab in bladder cancer, revealed that the levels of IGF2BP3 were notably elevated in the individuals who showed positive response (complete response/partial response) in contrast to those who did not respond (stable disease/progressive disease) (Fig. 7Q). Furthermore, increased levels of IGF2BP3 were associated with improved overall survival during anti-PD-L1 immunotherapy (Fig. 7Q). Analysis from GSE19423, a Bacillus Calmette–Guérin (BCG) immunotherapy of bladder cancer dataset, also revealed that a high level of IGF2BP3 was associated with favorable overall survival and cancer-specific survival in patients with bladder cancer (Fig. 7R). These findings suggest that IGF2BP3 might impact the immune microenvironment of bladder cancer through HMGB1, consequently impacting the efficacy of immunotherapy in bladder cancer.

Collectively, our results indicate that IGF2BP3 promotes HMGB1 stability by directly binding to HMGB1 mRNA through m6A modification in bladder cancer cells and suggest a potential role for IGF2BP3 in modulating the immune response in BLCA and its impact on immunotherapy efficacy.

### Glycyrrhizin reverses the effects of IGF2BP3 on bladder cancer progression through inhibition of HMGB1

**Fig. 8:**
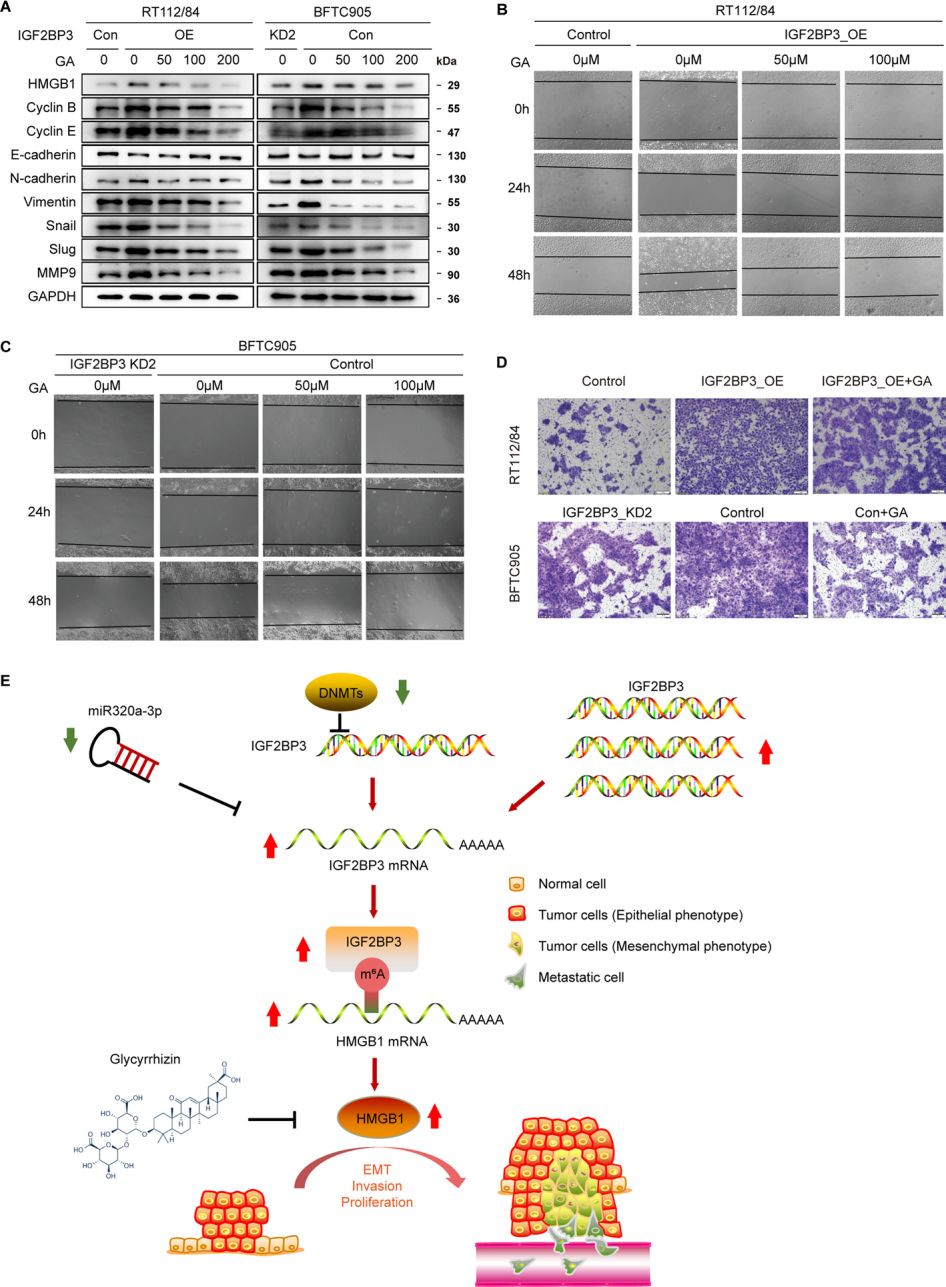
HMGB1 inhibitor glycyrrhizin reverses the cancer-promoting effect induced by IGF2BP3 overexpression. **A** Western blot analysis of protein levels in HMGB1, Cyclin B, Cyclin E, E-cadherin, N-cadherin, Vimentin, Snail, Slug, MMP9 in IGF2BP3-overexpression RT112/84 and BFTC905 cells treated with increasing concentrations of HMGB1 inhibitor glycyrrhizin (GA) for 24 h. **B**–**D** The effect of glycyrrhizin on cell migration and invasion of IGF2BP3-overexpression RT112/84 and BFTC905 cells was evaluated by wound-healing assay and transwell assay, respectively. **E** Schematic representation of the study

In order to confirm that the observed phenotypes were indeed caused by the aberrant IGF2BP3-HMGB1 pathway, we conducted restoration experiments by utilizing the HMGB1 inhibitor glycyrrhizin. Western blot demonstrated that glycyrrhizin treatment reduced the expression of Cyclin B and Cyclin E, which were induced by IGF2BP3 overexpression (Fig. 8A). This indicated that cell proliferation was significantly inhibited upon HMGB1 inhibition. Additionally, glycyrrhizin treatment attenuated the pro-EMT effect driven by IGF2BP3, as evidenced by the reduction in N-cadherin and vimentin expression, and the restoration of E-cadherin expression (Fig. 8A). Moreover, migration and invasion of BFTC905 and IGF2BP3-overexpressing RT112/84 cells were significantly suppressed upon incubation with glycyrrhizin (Fig. 8B–D).

These findings indicate that IGF2BP3 promotes cell migration and invasion by activating EMT through HMGB1, and that glycyrrhizin has the potential to be used as a therapeutic option for bladder cancer treatment (Fig. 8E).

### Interplay between IGF2BP3 and HMGB1 during development

The prevailing viewpoint suggests that there are significant similarities between embryonic development and tumorigenesis [64]. For instance, EMT, which was initially observed during embryonic development, is believed to play a crucial role in tumor invasion and metastasis [64, 65]. Additionally, inflammation has also shown numerous similarities with embryogenesis and tumorigenesis [64]. During tumor occurrence and progression, certain genes vital to embryonic development are re-expressed. Given the elevated expression of IGF2BP3 in various tumors, we postulate that IGF2BP3 likely exhibits abundant expression and a role in early embryonic development. To test this hypothesis, we conducted the following analyses.

**Fig. 9:**
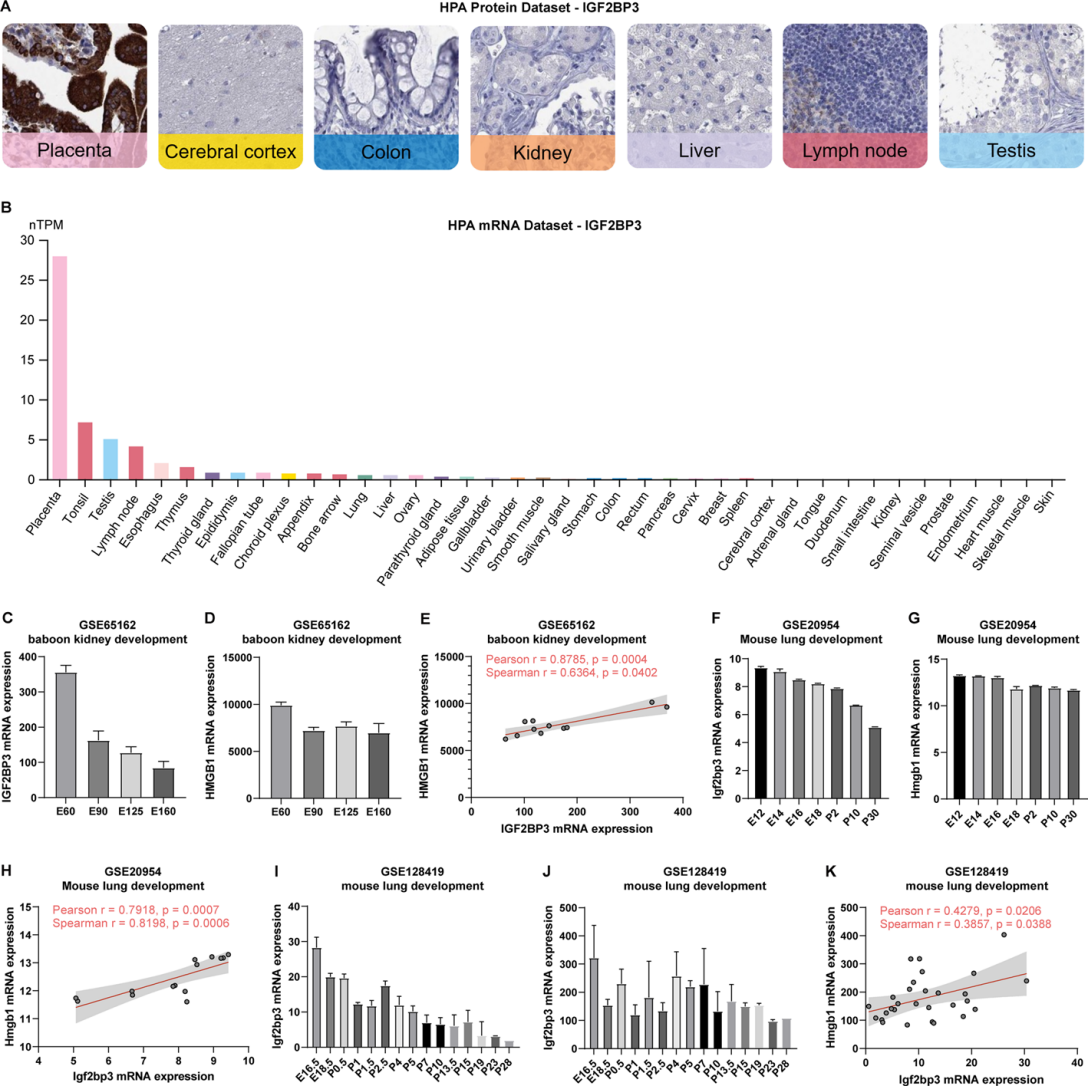
The relationship between IGF2BP3 and HMGB1 during embryonic development. **A** Representative IHC images of IGF2BP3 in various normal human tissues from the HPA database. **B** IGF2BP3 mRNA levels across all available tissues ordered by IGF2BP3 expression in the HPA database. **C**, **D** The mRNA levels of IGF2BP3 and HMGB1 genes at different time points of baboon kidney development in GSE65162 dataset. **E** Correlation of IGF2BP3 and HMGB1 mRNA levels in GSE65162 dataset. **F**, **G** The mRNA levels of IGF2BP3 and HMGB1 genes at different time points of mouse lung development in GSE0954 dataset. **H** Correlation of IGF2BP3 and HMGB1 mRNA levels in GSE0954 dataset. **I**, **J** The mRNA levels of IGF2BP3 and HMGB1 genes at different time points of mouse lung development in GSE128419 dataset. **K** Correlation of IGF2BP3 and HMGB1 mRNA levels in GSE128419 dataset. E: Embryonic day; P: post natal day

First, by analyzing the HPA protein IHC dataset, we found that among various human tissues, IGF2BP3 exhibited the highest expression in the placenta, the extra-embryonic tissues (https://www.proteinatlas.org/ENSG00000136231-IGF2BP3/tissue) (Fig. 9A, Additional file 4: Fig.S4). Similarly, mRNA data analysis from HPA revealed substantially higher levels of IGF2BP3 expression in placental tissue compared to other tissues (Fig. 9B). Subsequently, we examined three GEO datasets focused on embryonic development. These included a non-human primate dataset for embryonic kidney development (GSE65162) and two mouse lung development datasets (GSE20954 and GSE128419). Analysis of GSE65162 dataset demonstrated high expression of IGF2BP3 during the early stages of embryonic development, which gradually decreased over time. And analysis of the GSE128419 and GSE20954 datasets unveiled a gradual decrease in IGF2BP3 expression not only during embryonic development but also as mice aged after birth. Furthermore, our analysis of mRNA levels of the HMGB1 gene across these three datasets also suggested a progressive decline in HMGB1 expression during embryonic development. Notably, correlation analysis presented a strong positive association between HMGB1 and IGF2BP3 expression levels within all these datasets, further supporting our previous result that IGF2BP3 positively regulates HMGB1 expression.

In summary, our research demonstrates the high expression of IGF2BP3 during early embryonic development, with subsequent gradual decrease as embryonic development progresses and age. This implies a pivotal role for IGF2BP3 in early mammalian development. Furthermore, IGF2BP3 significantly influences HMGB1 expression throughout development.

## Discussion

Recent findings indicate that the involvement of IGF2BP1/2/3, known as m6A readers, in the regulation of RNA stability plays a crucial role in tumor initiation, progression, and evolution. This disruption of normal expression and critical cellular signaling bioprocesses, including oncogenes and tumor suppressors, ultimately leads to the development and advancement of tumorigenesis [66–68]. In the present study, we identified a crucial regulatory network involving IGF2BP3 and altered HMGB1 through m6A modulation pattern, which is associated with BLCA progression. The expression of IGF2BP3 was elevated in BLCA tissues compared to adjacent normal tissues, and this increased level was correlated with advanced characteristics of BLCA and unfavorable prognosis in BLCA patients. Furthermore, the overexpression of IGF2BP3 in BLCA was attributed to the amplification/gain of IGF2BP3 copy number, the hypomethylation of its promoter, and the deficiency of miR-320a-3p. IGF2BP3 could significantly enhance the capacity of proliferation, migration, and invasion of BLCA cells. IGF2BP3, an RNA-binding protein, identifies and selectively binds to the mRNA of HMGB1, resulting in the increased expression of both HMGB1 mRNA and protein. We also showed that glycyrrhizin, an HMGB1 antagonist, could inhibit BLCA progression. Consequently, our discoveries yielded a range of new biomarkers and targets concerning epigenetic alteration for the purpose of predicting diagnosis, evaluating clinical outcomes, and implementing targeted treatment of bladder cancer.

Numerous studies, including our own, have shown that IGF2BP3 is a cancer-causing factor and frequently exhibits high levels of expression in various types of cancer [68]. In a recent study, it was found that circNFATC3 has the ability to attach to IGF2BP3, resulting in an increase in the stability of IGF2BP3 by obstructing the ubiquitin E3 ligase TRIM25 from causing ubiquitination. As a result, this enhances the expression of IGF2BP3 [69]. Nevertheless, the precise molecular mechanism governing the regulation of IGF2BP3 expression remains largely unknown. CNA can regulate gene expression through gene dosage, and then promote tumor progression by altering gene expression levels [70]. For example, most (> 90%) of LSCC (Lung Squamous Cell Carcinoma) tumors exhibit an increase in copy number at chromosome 3q26, resulting in the upregulation of the PRKCI, SOX2, and ECT2 oncogenes and LSCC tumorigenesis [71]. In a previous study from our group, we found that 1p/19q codeletion in LGGs (low-grade glioma) could decrease the expression of more than 100 immune-related genes and then affect TME and the prognosis of LGGs [72]. Another study of our group demonstrated that copy number loss of SPNS2 in colorectal cancer led to low expression of SPNS2, which inhibits metastasis of colorectal cancer [73]. In the present study, we found that more than 45% of bladder cancer harbor copy number gain/amplification of IGF2BP3, and its copy number positively correlated with IGF2BP3 expression. This indicated that copy number variation is one of key regulator of IGF2BP3 expression. Moreover, we found that IGF2BP3 expression positively correlated with its copy number in 22 kinds of tumor through analyzing the multi-omics data of TCGA cohort. This suggests that copy number gain/amplification contribute to IGF2BP3 upregulation in a wide-variety of tumor.

In contrast to the positive regulation of gene copy number on IGF2BP3 expression, our current study provides evidence that promoter methylation serves as an inhibitory factor for IGF2BP3 expression in bladder cancer. Furthermore, our pan-cancer analysis revealed that the methylation status of IGF2BP3 promoter negatively correlated with its expression in various tumor types, indicating that the methylation regulation of IGF2BP3 expression is also present in other tumors. In fact, numerous previous studies have corroborated the significant role that promoter methylation plays in impeding gene transcription [74]. For instance, our recent research demonstrated that hypermethylation of the TRIM29 promoter led to its downregulation, thereby promoting tumor metastasis in esophageal squamous cell carcinoma [43]. Similarly, hypermethylation of the HSD17B6 promoter was shown to decrease its expression, contributing to the progression of hepatocellular carcinoma [42]. Promoter methylation also serves as a crucial regulator of NLRC5 expression, a key player in antigen presentation and inflammation [75]. Our other recent findings have showcased the predominant expression of ACAP1 in lymphocytes and its indispensable role in maintaining normal lymphocyte function. The ACAP1 promoter is hypomethylated in immune tissues and lymphocytes, though it is heavily methylated in other tissues and cells [76]. DNA methylation inhibits the transcription process primarily by disrupting the interaction between DNA elements and transcription factors [77]. Therefore, further research is needed to reveal which transcription factors mainly regulate the expression of IGF2BP3 in the future.

The other significant finding in this study is that HMGB1 is a novel m6A-modified target that can be recognized by IGF2BP3, leading to increased stability of HMGB1. HMGB1, one member of the HMGB family, plays a role in diverse physiological and pathological cellular processes, such as cell proliferation, migration, and differentiation. It plays a pivotal role in inflammation, a characteristic of carcinogenesis, and could cause changes in the microenvironment, thereby impacting the development of carcinoma [78]. For example, HMGB1 secretion recruits inflammatory cells, leading to the development of cancer in mesothelial cells [79]. HMGB1 could also influence tumor microenvironment to regulate tumor progression [80, 81]. The present research revealed that expression of IGF2BP3, acting as a positive regulator of HMGB1, exhibited a positive correlation with the degree of immune cell infiltration in bladder cancer. And high IGF2BP3 levels were related to good prognosis in immunotherapy of bladder cancer. It is worth noting that this result is opposite to the situation under non-immunotherapy conditions. This demonstrates the dual role of IGF2BP3 and HMGB1 in tumor development and treatment. Further in-depth research is required to clarify the specific mechanisms involved.

HMGB1 could trigger autophagy through multiple pathways [82], which is crucial for enhancing cell viability by gathering proteins and removing impaired organelles through lysosomal degradation. For example, HMGB1 could bind RAGE to stimulate Beclin-1-dependent autophagy [83]. Additionally, HMGB1 might have a notable impact on apoptosis by inhibiting pro-apoptotic molecules like caspase-3 and BAX, or by enhancing the expression of antiapoptotic molecules like Bcl-2 [84]. Furthermore, it can stimulate the expression of neuropilin-1, endothelial growth factor A (VEGFA), and VEGF receptors 1 and 2, thereby triggering angiogenesis [85]. Furthermore, apart from these biological processes related to tumorigenesis, HMGB1 is also involved in tumor invasion and metastasis [86], which are the foremost distinguishing features of malignant neoplasms. In a RAGE-dependent manner, HMGB1 has the ability to trigger EMT by increasing the levels of various proteins that function as inducers of EMT, such as MMP-7, phosphorylated NF-kB, and Snail [87]. HMGB1/RAGE axis and EMT have also been linked to the generation of NF-kB/p65, iNOS, MMP9, and the activation of Rac1, ERK 1/2, and AKT [88]. Our current research revealed that IGF2BP3 could increase the expression of HMGB1 and promote bladder cell EMT and invasion. And inhibition of HMGB1 with glycyrrhizin could reverse these effects. Our findings, combined with these previous studies, showed that HMGB1 is an important intermediate molecule of IGF2BP3 induced tumor EMT and invasion/metastasis. Therefore, it came to the conclusion that IGF2BP3 activated the HMGB1 related pathways and participated in tumor progression of BLCA.

## Conclusions

To summarize, our investigations have shed light on the mechanism by which IGF2BP3 functions in BLCA and development. Nevertheless, there is still a wealth of knowledge yet to uncover regarding the underlying mechanism, such as the specific binding site of IGF2BP3 on the HMGB1 mRNA. Nevertheless, our study: (1) revealed the regulatory mechanism of IGF2BP3 expression as well as the cause of its abnormal expression; (2) confirmed that IGF2BP3 can promote bladder cancer development as well as revealed its molecular mechanism; (3) revealed molecular mechanism by which IGF2BP3 regulates the expression of HMGB1; and (4) demonstrated that IGF2BP3-HMGB1 can serve as a bladder cancer therapeutic target and the potential of glycyrrhizin as a therapeutic agent for bladder cancer. These findings could contribute to improved diagnosis, prognosis prediction, and personalized treatment decisions.

### Supplementary Information

**Additional file 1:** **Figure S1. **The expression of IGF2BP1/2/3 and their prognostic value in bladder cancer of TCGA BLCA dataset.

**Additional file 2:Figure S2.** Analysis of IGF2BP3 expression in other bladder cancer datasets.

**Additional file 3:** **Figure S3.** The percentage of apoptotic cells was determined by flow cytometric analysis.

**Additional file 4:** **Figure S4.** IGF2BP3 protein expression levels across all available tissues ordered by IGF2BP3 expression in the HPA Portal.

**Additional file 5:** **Table S1.** List of common HALLMARKs significantly correlated with IGF2BP3 expression in TCGA BLCA, E-MATB-4321, and GSE48276 datasets.

**Additional file 6: Table S2.** POSTAR3 Binding site records of target HMGB1 RNA.

## Data Availability

Data are available from the corresponding author upon reasonable request.

## Abbreviations

TCGA: The Cancer Genome Atlas
GEO: Gene Expression Omnibus
HPA: The Human Protein Atlas
BLCA: Bladder urothelial carcinoma
RBP: RNA-binding protein
EMT: Epithelial–mesenchymal transition
CNA: Copy number alteration
3′-UTR: 3′-Untranslated region
OS: Overall survival
DSS: Disease specific survival
PFI: Progression free interval
RFS: Recurrence free survival
GSEA: Gene Set Enrichment Analysis
TIMER: Tumor Immune Estimation Resource
BCG: Bacillus Calmette–Guérin
VEGF: Endothelial growth factor
