# Supplementary material for: IGF2BP3 prevent HMGB1 mRNA decay in bladder cancer and development
Source: Cell Mol Biol Lett. 2024 Mar 19;29:39. doi: 10.1186/s11658-024-00545-1 (PMC10949762; doi:10.1186/s11658-024-00545-1)
Supplement: Supplementary file 1 — Additional file 1: Figure S1. The expression of IGF2BP1/2/3 and their prognostic value in bladder cancer of TCGA BLCA dataset. [file 11658_2024_545_MOESM1_ESM.docx]

**
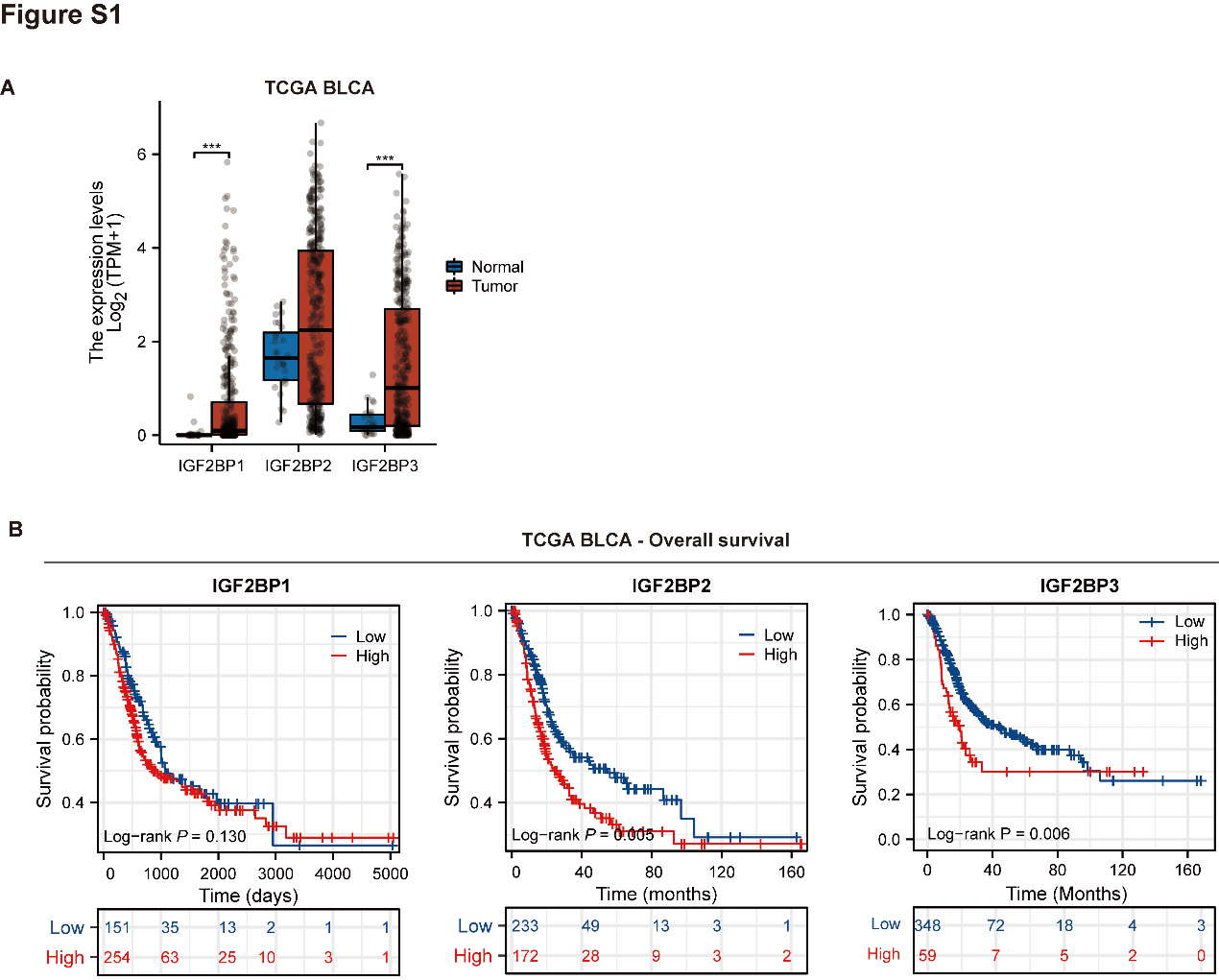
**

**Figure S1. The expression of IGF2BP1/2/3 and their prognostic value in bladder cancer of TCGA BLCA dataset.** (A) Comparison of IGF2BP1/2/3 mRNA levels between tumor and normal tissue in TCGA BLCA. (B) Kaplan-Meier analysis of OS (Overall survival) based on IGF2BP1/2/3 expression for bladder cancer patients in the TCGA BLCA dataset.
