# Supplementary material for: IGF2BP3 prevent HMGB1 mRNA decay in bladder cancer and development
Source: Cell Mol Biol Lett. 2024 Mar 19;29:39. doi: 10.1186/s11658-024-00545-1 (PMC10949762; doi:10.1186/s11658-024-00545-1)
Supplement: Supplementary file 2 — Additional file 2:Figure S2. Analysis of IGF2BP3 expression in other bladder cancer datasets. [file 11658_2024_545_MOESM2_ESM.docx]

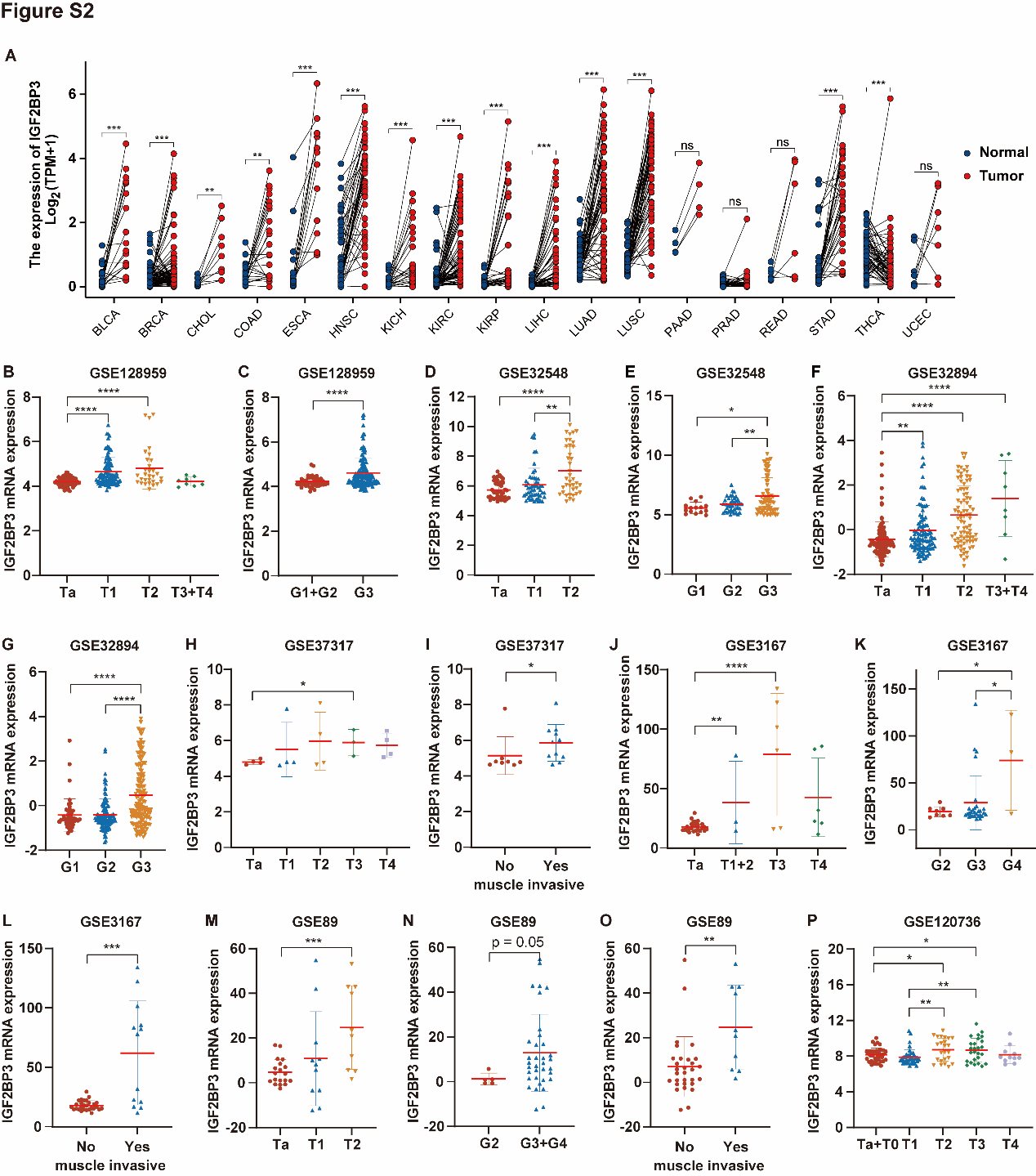


**Figure S2. Analysis of IGF2BP3 expression in other bladder cancer datasets**. (**A**) Paired comparison of IGF2BP3 mRNA levels between tumor and normal tissue across TCGA cancer types in TCGA datasets. (**B-P**) Comparison of IGF2BP3 mRNA levels in different T stage, Grade, invasive status in TCGA BLCA in multiple bladder cancer datasets. P-value Significant Codes: **** < 0.0001, *** < 0.001, ** < 0.01, * < 0.05.
