# Supplementary figures and images for: IGF2BP3 prevent HMGB1 mRNA decay in bladder cancer and development
Source: Cell Mol Biol Lett. 2024 Mar 19;29:39. doi: 10.1186/s11658-024-00545-1 (PMC10949762; doi:10.1186/s11658-024-00545-1)

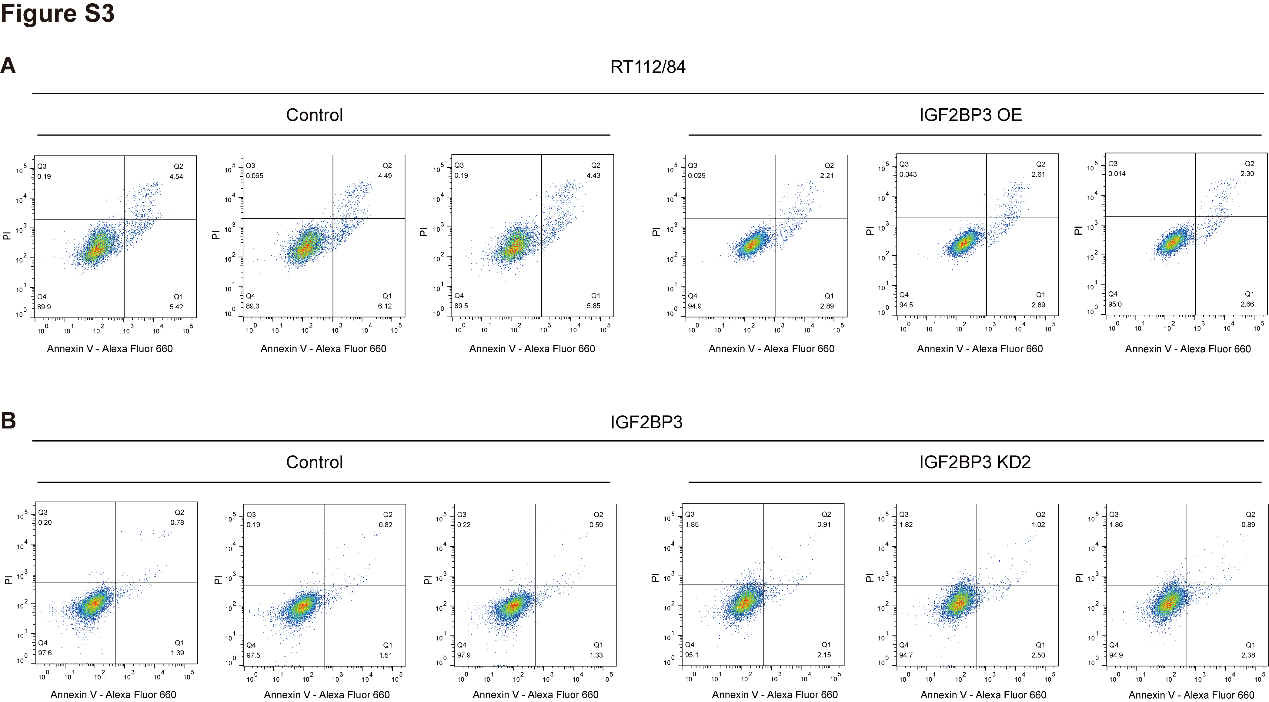


**Figure S3. The percentage of apoptotic cells was determined by flow cytometric analysis.**

Supplement: Supplementary file 3 — Additional file 3: Figure S3. The percentage of apoptotic cells was determined by flow cytometric analysis. [file 11658_2024_545_MOESM3_ESM.docx]
