# Supplementary material for: IGF2BP3 prevent HMGB1 mRNA decay in bladder cancer and development
Source: Cell Mol Biol Lett. 2024 Mar 19;29:39. doi: 10.1186/s11658-024-00545-1 (PMC10949762; doi:10.1186/s11658-024-00545-1)
Supplement: Supplementary file 4 — Additional file 4: Figure S4. IGF2BP3 protein expression levels across all available tissues ordered by IGF2BP3 expression in the HPA Portal. [file 11658_2024_545_MOESM4_ESM.docx]

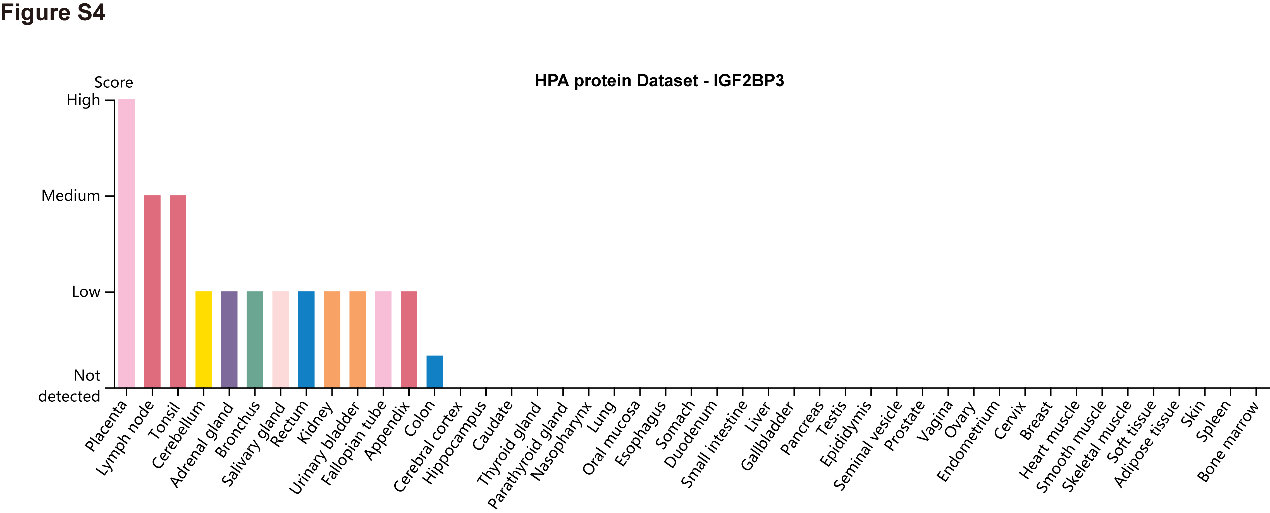


**Figure S4. IGF2BP3 protein levels across all available tissues ordered by IGF2BP3 expression in the HPA database.**
